# A LlWRKY33-LlHSFA4-LlCAT2 module confers resistance to *Botrytis cinerea* in lily

**DOI:** 10.1093/hr/uhad254

**Published:** 2023-11-27

**Authors:** Liping Ding, Ze Wu, Jun Xiang, Xing Cao, Sujuan Xu, Yinyi Zhang, Dehua Zhang, Nianjun Teng

**Affiliations:** Key Laboratory of Landscaping, Ministry of Agriculture and Rural Affairs, Key Laboratory of Biology of Ornamental Plants in East China, National Forestry and Grassland Administration, College of Horticulture, Nanjing Agricultural University, Nanjing 210095, China; Jiangsu Graduate Workstation of Nanjing Agricultural University and Nanjing Oriole Island Modern Agricultural Development Co., Ltd., Nanjing 210043, China; Key Laboratory of Landscaping, Ministry of Agriculture and Rural Affairs, Key Laboratory of Biology of Ornamental Plants in East China, National Forestry and Grassland Administration, College of Horticulture, Nanjing Agricultural University, Nanjing 210095, China; Jiangsu Graduate Workstation of Nanjing Agricultural University and Nanjing Oriole Island Modern Agricultural Development Co., Ltd., Nanjing 210043, China; Key Laboratory of Landscaping, Ministry of Agriculture and Rural Affairs, Key Laboratory of Biology of Ornamental Plants in East China, National Forestry and Grassland Administration, College of Horticulture, Nanjing Agricultural University, Nanjing 210095, China; Jiangsu Graduate Workstation of Nanjing Agricultural University and Nanjing Oriole Island Modern Agricultural Development Co., Ltd., Nanjing 210043, China; College of Architecture, Yantai University, Yantai, 264005, China; Key Laboratory of Landscaping, Ministry of Agriculture and Rural Affairs, Key Laboratory of Biology of Ornamental Plants in East China, National Forestry and Grassland Administration, College of Horticulture, Nanjing Agricultural University, Nanjing 210095, China; Jiangsu Graduate Workstation of Nanjing Agricultural University and Nanjing Oriole Island Modern Agricultural Development Co., Ltd., Nanjing 210043, China; Key Laboratory of Landscaping, Ministry of Agriculture and Rural Affairs, Key Laboratory of Biology of Ornamental Plants in East China, National Forestry and Grassland Administration, College of Horticulture, Nanjing Agricultural University, Nanjing 210095, China; Jiangsu Graduate Workstation of Nanjing Agricultural University and Nanjing Oriole Island Modern Agricultural Development Co., Ltd., Nanjing 210043, China; Key Laboratory of Landscaping, Ministry of Agriculture and Rural Affairs, Key Laboratory of Biology of Ornamental Plants in East China, National Forestry and Grassland Administration, College of Horticulture, Nanjing Agricultural University, Nanjing 210095, China; Jiangsu Graduate Workstation of Nanjing Agricultural University and Nanjing Oriole Island Modern Agricultural Development Co., Ltd., Nanjing 210043, China; Key Laboratory of Landscaping, Ministry of Agriculture and Rural Affairs, Key Laboratory of Biology of Ornamental Plants in East China, National Forestry and Grassland Administration, College of Horticulture, Nanjing Agricultural University, Nanjing 210095, China; Jiangsu Graduate Workstation of Nanjing Agricultural University and Nanjing Oriole Island Modern Agricultural Development Co., Ltd., Nanjing 210043, China

## Abstract

Gray mold caused by *Botrytis cinerea* is one of the major threats in lily production. However, limited information is available about the underlying defense mechanism against *B. cinerea* in lily. Here, we characterized a nuclear-localized class A heat stress transcription factor (HSF)-LlHSFA4 from lily (*Lilium longiflorum*), which positively regulated the response to *B. cinerea* infection. *LlHSFA4* transcript and its promoter activity were increased by *B. cinerea* infection in lily, indicating its involvement in the response to *B. cinerea*. Virus-induced gene silencing (VIGS) of *LlHSFA4* impaired the resistance of lily to *B. cinerea*. Consistent with its role in lily, overexpression of *LlHSFA4* in Arabidopsis (*Arabidopsis thaliana*) enhanced the resistance of transgenic Arabidopsis to *B. cinerea* infection. Further analysis showed that LlWRKY33 directly activated *LlHSFA4* expression. We also found that both LlHSFA4 and LlWRKY33 positively regulated plant response to *B. cinerea* through reducing cell death and H_2_O_2_ accumulation and activating the expression of the reactive oxygen species (ROS) scavenging enzyme gene *LlCAT2* (*Catalase 2*) by binding its prompter, which might contribute to reducing H_2_O_2_ accumulation in the infected area. Taken together, our data suggested that there may be a LlWRKY33-LlHSFA4-LlCAT2 regulatory module which confers *B. cinerea* resistance via reducing cell death and the ROS accumulation.

## Introduction

Necrotrophic fungi kill host cells and infect host plants by secreting keratase, cell wall degrading enzymes, phytotoxic metabolites, and other pathogenic factors in host plants and subsequently absorb nutrients from dead cells of the host and grow and expand rapidly [1]. *Botrytis cinerea* is a typical necrotrophic fungal pathogen, which infects a broad range of plants including fruits, vegetables, flowers, causing huge economic losses in the world every year [2]. Through the co-evolution of plants and their potential necrotrophic pathogens, plants have evolved complex strategies to defend against these pathogens, including regulation of hormone-signaling pathways, production of antimicrobial metabolites, and transcription factor (TF)-mediated transcriptional regulation to protect themselves from necrotrophic pathogens [3–5]. TF-mediated transcriptional regulation is a critical step to activate plant immune response [6–8].

HSFs exist widely in plants and are classified into three classes, HSFA, HSFB, and HSFC, based on the structural characteristics [9]. Typically, HSFs, especially HSFAs which are intensively studied in abiotic stress, have been found to play roles in abiotic stress by binding heat shock elements (HSEs) in the promoters of their targeted gene to regulate abiotic stresses, including heat [10–12], drought [13], salinity [14], and oxidative damage response [15]. There is evidence that HSFBs could participate in the response to pathogen attacks. HSFB1 was reported to suppress the innate immune response of Arabidopsis and could also be involved in the defense initiation and systemic acquired resistance in Arabidopsis [16, 17]. *Oryza sativa* HSFB4d directly binds to the HSE in the promoter of *OsHsp18.0-CI* to defend against *Xanthomonas oryzae* [18]. However, the function of HSFAs in the plant’s response to pathogen remains poorly understood.

In different plants, many studies have shown that WRKY33 is a key positive regulator for host immunity to *B. cinerea*. In Arabidopsis, WRKY33 was directly phosphorylated by MAP kinases after *B. cinerea* infection and activated camalexin biosynthesis gene *PAD3* expression [19, 20]. Arabidopsis WRKY33 was also required for the hormonal signaling and metabolic responses to *B. cinerea* infection [21]. WRKY33 decreased levels of abscisic acid (ABA) by directly repressing the expression of *NCED3* and *NCED5* during *B. cinerea* infection [22]. In tomato (*Solanum lycopersicum*), *WRKY33* homologues (*SlWRKY33A* and *SlWRKY33B*) can restore the resistance of Arabidopsis *wrky33* mutant plants to *B. cinerea* [23]. However, it was still unclear whether HSFs are involved in the regulatory network of WRKY33.

In this study, we report the functional characterization of *LlHSFA4* and *LlWRKY33* following inoculation with *B. cinerea* spores. Both LlHSFA4 and LlWRKY33 contribute to defense against *B. cinerea* by reducing cell death and the ROS accumulation in the infected area. Further studies indicate that LlWRKY33 binds the W-box element on the promoters of *LlHSFA4* and *LlCAT2* and activates their expression. These results imply that LlHSFA4 confers *B. cinerea* resistance via forming transcriptional cascades with *LlWRKY33* and *LlCAT2*.

## Results

###  LlHSFA4 is a *B. cinerea*-inducible HSFA in lily

**Figure 1:**
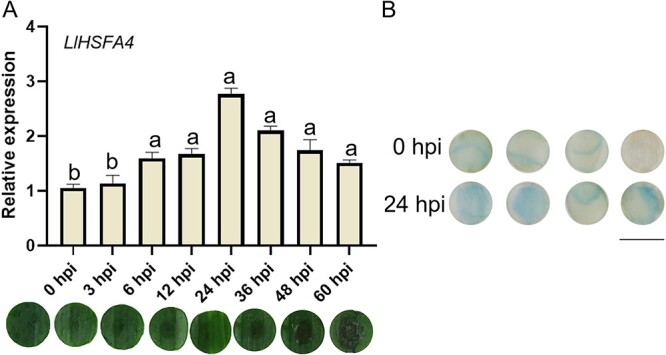
*LlHSFA4* is induced by *Botrytis cinerea*. **A** The expression levels of *LlHSFA4* in lily leaves after *B. cinerea* infection at various hours post inoculation. Disease lesion of leaf discs following inoculation with different hours was shown under the bar graph. Different letters indicate significant differences determined by Tukey’s test with the standard deviation (P < 0.05). **B**. Analysis of the promoter activity of *LlHSFA4* in lily petal discs at 0 hpi and 24 hpi. Scale bar = 1 cm.

To determine the expression patterns of *LlHSFAs* (*LlHSFA1*, *LlHSFA2*, *LlHSFA3A*, and *LlHSFA4*) under *B. cinerea* infection, the detached leaf discs of *Lilium longiflorum* ‘White Heaven’ were chosen to be inoculated with *B. cinerea* spores. The RT-qPCR results implicated that *LlHSFA4* was the only inducible *HSFA* gene compared with control plants, while the expression of *LlHSFA1*, *LlHSFA2*, and *LlHSFA3A* was not induced by *B. cinerea* (Fig. 1A; Fig. S1, see online supplementary material). Notably, the necrotic lesions were obviously observed on infected leaves at 36 hours post inoculation (hpi) (Fig. 1A), which implicated cell death occurs between 24 hpi and 36 hpi in the infected leaves. Next, we isolated 1500-bp promoter region of *LlHSFA4* and identified its activity after *B. cinerea* infection. With an online promoter analysis, several *cis*-acting elements were present in *LlHSFA4* promoter, including W-box element, low temperature responsive element (LTRE), R response element (RRE), ABA response element (ABRE), and others, which implicated that LlHSFA4 might participate in response to biotic or abiotic stress (Table S1, see online supplementary material). Then, a *proLlHSFA4*-GUS vector was constructed and transformed into lily to determine the promoter activity of *LlHSFA4* after *B. cinerea* inoculation. The inoculated lily petal discs were subjected to histochemical staining for GUS activity after 0 hpi and 24 hpi. The blue color observed in lily petal discs indicates the active expression of *LlHSFA4* gene. It was found that the lily petal discs at 24 hpi appeared to have a deeper blue color than those at 0 hpi, indicating that the promoter activity of *LlHSFA4* could be activated by *B. cinerea* (Fig. 1B). These data indicate *LlHSFA4* is induced by *B. cinerea*.

### Silencing of *LlHSFA4* increases susceptibility of lily to *B. cinerea*

**Figure 2:**
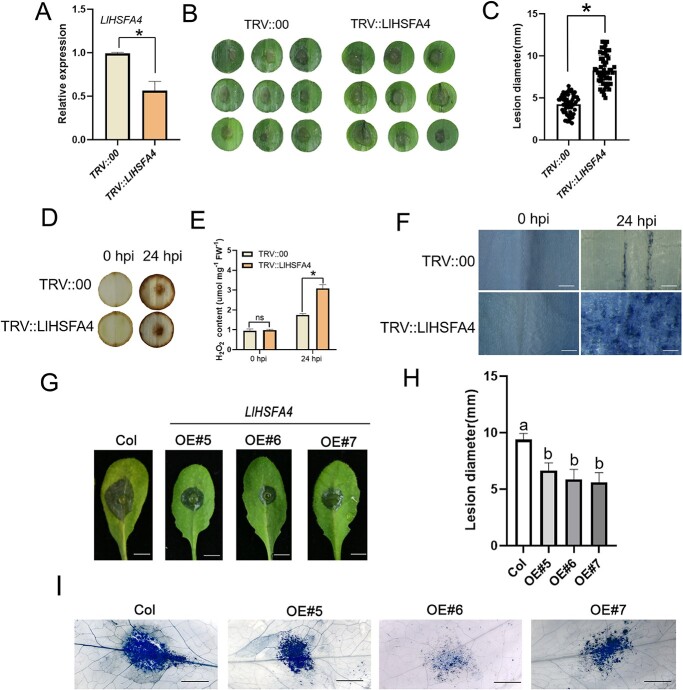
LlHSFA4 positively regulates plant response to *Botrytis cinerea*. **A** The silencing efficiency of *LlHSFA4* in TRV::*LlHSFA4* plants detected by RT-qPCR. Significant differences are presented as means ± SD of three replicates (Student’s *t* test, ^*^P < 0.05). **B** Disease symptoms of TRV::00 and TRV::*LlHSFA4* plants at 60 hpi by *B. cinerea* droplet-inoculation. **C** Lesion diameter of TRV::00 and TRV:: *LlHSFA4* at 60 hpi by *B. cinerea* droplet-inoculation. The graph shows the average lesion size from three biological replicates (*n* = 60). Bars are determined by student’s *t* test with the standard deviation (^*^P < 0.05). **D** DAB staining of H_2_O_2_ of TRV::00 and TRV::*LlHSFA4* leaf discs at 0 hpi and 24 hpi. **E** The determination of H_2_O_2_ content of TRV::00 and TRV::*LlHSFA4* leaf discs at 0 hpi and 24 hpi. ns: not significant. **F** Trypan blue staining of TRV::00 and TRV::*LlHSFA4* plants at 0 hpi and 24 hpi. Scale bar = 500 μm. **G** Disease symptoms of transgenic Arabidopsis (OE-5, OE-6, and OE-7) at 48 hpi by *B. cinerea* droplet-inoculation. Scale bar = 5 mm. **H** Lesion diameter of rosette leaves of the *LlHSFA4*-transgenic Arabidopsis at 48 hpi. Different letters indicate significant differences determined by Tukey’s test with the standard deviation (P < 0.05). The graph shows the average lesion size from three biological replicates (*n* = 30). **I** Trypan blue staining of rosette leaves of the *LlHSFA4*-transgenic Arabidopsis at 48 hpi. Scale bar = 2 mm.

To further confirm the function of *LlHSFA4* in the response to *B. cinerea*, we performed tobacco rattle virus (TRV)-mediated VIGS of *LlHSFA4* in lily leaves. The 5-day infiltrated leaves were harvested to test the silencing efficiency of *LlHSFA4*. And the results indicated that the *LlHSFA4* was effectively silenced in lily plants (Fig. 2A). After *B. cinerea* inoculation, the *LlHSFA4*-silenced lily plants showed more severe disease symptoms than VIGS-controls (Fig. 2B and C). Then, we determined the H_2_O_2_ accumulation at 24 hpi in the infected leaves of *LlHSFA4*-silenced leaves. The results showed that the infected leaves of *LlHSFA4*-silenced plants had a high level of H_2_O_2_ accumulation at 24 hpi than that of the VIGS-controls (Fig. 2D and E), suggesting that more ROS was accumulated in *LlHSFA4*-silencing leaves during *B. cinerea* infection. In addition, the results of microscopical observation of trypan blue staining showed that more dead cells were clearly visible at 24 hpi in *LlHSFA4*-silenced leaves than in VIGS-control leaves (Fig. 2F). All these results implicated that silencing of *LlHSFA4* increased the susceptibility of lily to *B. cinerea*.

### Overexpression of *LlHSFA4* improves the resistance of transgenic Arabidopsis to *B. cinerea*

The three *LlHSFA4*-transgeniclines (OE-5, OE-6, and OE-7) were confirmed by RT-PCR (Fig. S2, see online supplementary material). The wild-type and three *LlHSFA4*-transgenic lines were inoculated with *B. cinerea* using droplet-inoculation or spray-inoculation to explore the role of *LlHSFA4* under *B. cinerea* infection. At 48 hpi, the necrotic disease symptoms of wild-type were more severe than that of the transgenic lines (Fig. 2G and H; Fig. S3, see online supplementary material). Trypan blue staining implicated that overexpression of *LlHSFA4* decelerated the cell death in transgenic Arabidopsis (Fig. 2I).

###  LlWRKY33 directly activates the expression of *LlHSFA4* by binding its promoter

**Figure 3:**
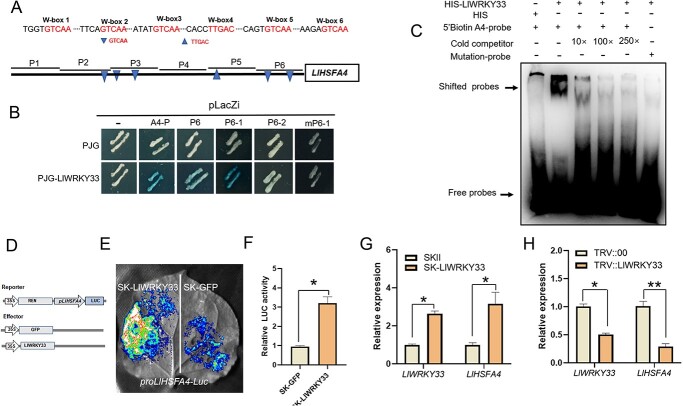
LlWRKY33 activates the expression of *LlHSFA4*. **A***LlHSFA4* promoter and W-box elements marked with blue triangles. **B** Y1H assay for LlWRKY33 and *LlHSFA4* promoter. **C** EMSA of HIS-LlWRKY33 and the W-box element. **D** Schematic representation of the dual-luciferase reporter assay. **E** LUC signal in *N. benthamiana* leaves. Representative image was from one of three biological replicates. **F** Measurement of relative LUC activity. Significant differences are presented as means ± SD of three replicates (Student’s *t* test, ^*^P < 0.05). **G** The expression of *LlWRKY33* and *LlHSFA4* in *LlWRKY33*-overexpressing lily plants. Significant differences are presented as means ± SD of three replicates (Student’s *t* test, ^*^P < 0.05). **H** The expression of *LlWRKY33* and *LlHSFA4* in *LlWRKY33*-silencing lily plants. Significant differences are presented as means ± SD of three replicates (Student’s *t* test, ^*^P < 0.05, ^**^P < 0.01). Three biological replicates were performed for each experiment.

It was found that six typical W-box elements were present in the promoter of *LlHSFA4* (Fig. 3A). The WRKY transcription factor WRKY33 has been shown to act as a critical regulator in the response to *B. cinerea* infection [19–25]. Therefore, we speculated that *LlHSFA4* might be transcriptionally modulated by LlWRKY33.Yeast one-hybrid assay implicated that LlWRKY33 could directly bind the A4-P and only bind the truncated P6 fragment of *LlHSFA4* promoter (Fig. 3B; Fig. S4, see online supplementary material). Promoter analysis of the A4-P6 fragment revealed that two W-box elements were present in the P6 fragment (Fig. 3A). Then, the A4-P6 fragment was further truncated into two fragments (A4-P6–1, A4-P6–2) for a Y1H assay (Fig. 3B). Further Y1H assay implicated that LlWRKY33 directly bound the W-box element located at −285 bp to −280 bp (GTCAA) on the A4-P6–1fragment, but not A4-P6–2 and muted A4-P6–1 fragment in which W-box elements (GTCAA) was mutated (TTTTT) (Fig. 3B). Based on the results of the Y1H assays, the promoter region containing W-box element of A4-P6–1fragment was synthesized into a 5′ biotin-labeled probe for performing an electrophoretic mobility shift assay. The result of EMSA was consistent with those of Y1H (Fig. 3C). LUC activity of *Nicotiana benthamiana* leaves co-transformed with LlWRKY33 and *proLlHSFA4*-LUC was stronger than that of the SK-GFP and *proLlHSFA4*-LUC (Fig. 3E and F). Transient overexpression of *LlWRKY33* also activated the transcription of *LlHSFA4* in lily, while silencing of *LlWRKY33* reduced its expression (Fig. 3G and H). Therefore, these results suggested that LlWRKY33 could serve as a direct regulator of *LlHSFA4* and activate its expression.

### Silencing of *LlWRKY33* increases susceptibility of lily to *B. cinerea*

**Figure 4:**
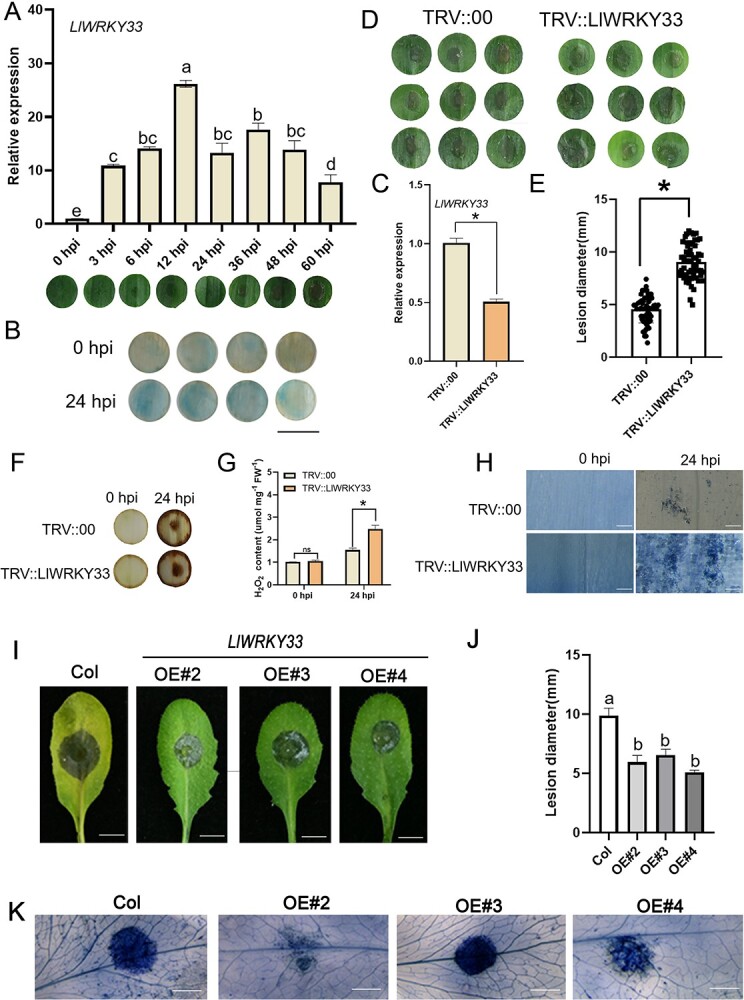
LlWRKY33 positively regulates plant response to *Botrytis cinerea*. **A** The expression of *LlWRKY33* in lily after *B. cinerea* infection at various hours post-inoculation. Disease lesion of leaf discs following inoculation with different hours was shown under the bar graph. Different letters indicate significant differences determined by Tukey’s test with the standard deviation (*P* < 0.05). **B** Analysis of *LlWRKY33* promoter activity in the lily petal discs. Scale bar = 1 cm. **C** The silencing efficiency of *LlWRKY33* in TRV::*LlWRKY33* plants detected by RT-qPCR. Significant differences are presented as means ± SD of three replicates (Student’s *t* test, *P < 0.05). **D** Disease symptoms of TRV::00 and TRV::*LlWRKY33* plants at 60 hpi by droplet-inoculation. **E** Lesion diameter of TRV::00 and TRV:: *LlWRKY33* plants at 60 hpi. The graph shows the average lesion size from three biological replicates (*n* = 60). Significant differences are presented as means ± SD of three biological replicates (Student’s *t* test, ^*^P < 0.05). **F** DAB staining of H_2_O_2_ accumulation of TRV::00 and TRV::*LlWRKY33* plants at 0 hpi and 24 hpi. **G** Determination of H_2_O_2_ content of TRV::00 and TRV::*LlWRKY33* plants at 0 hpi and 24 hpi. Significant differences are presented as means ± SD of three replicates (Student’s *t* test, ^*^P < 0.05). ns: not significant. **H** Trypan blue staining of TRV::00 and TRV::*LlWRKY33* plants at 0 hpi and 24 hpi. Scale bar = 500 μm. **I** Disease symptoms of rosette leaves from the *LlHSFA4*-transgenic Arabidopsis (OE-2, OE-3, and OE-4) at 48 hpi by droplet-inoculation. Scale bar = 5 mm. **J** Lesion diameter of rosette leaves of the *LlHSFA4*-transgenic Arabidopsis at 48 hpi. Different letters indicate significant differences determined by Tukey’s test with the standard deviation (*P* < 0.05). The graph shows the average lesion size from three biological replicates (*n* = 30). **K** Trypan blue staining of rosette leaves from the *LlHSFA4*-transgenic Arabidopsis at 48 hpi. Scale bar = 2 mm. Three biological replicates were performed for each experiment.

The LlWRKY33-GFP signal and the nuclear marker were co-localized in the nucleus in *N. benthamiana* leaves, indicating that LlWRKY33 was a nucleus-localized protein (Fig. S5A, see online supplementary material). The results of transactivation activity showed that LlWRKY33 had transcriptional activation in yeast cells (Fig. S5B, see online supplementary material). To verify the function of *LlWRKY33* in the response to *B. cinerea*, we firstly performed RT-qPCR assay to examine the expression of *LlWRKY33* in lily leaves after *B. cinerea* infection. The results showed that *LlWRKY33*’s transcription was continuously elevated after *B. cinerea* infection and the highest expression level (26-fold induction) was at 12 hpi (Fig. 4A). Next, a 521-bp promoter of *LlWRKY33* was isolated and identified. Then, a *proLlWRKY33*-GUS was constructed and transformed into lily to determine the promoter activity of *LlWRKY33* after *B. cinerea* inoculation. The *proLlWRKY33*-GUS activity was higher in lily at 24 hpi than its level at 0 hpi, indicating that the promoter activity of *LlWRKY33* could be activated by *B. cinerea* (Fig. 4B). Then, we performed a VIGS experiment in lily. The results showed that *LlWRKY33* expression was effectively silenced in *LlWRKY33*-silenced lily plants compared with TRV2-controls (Fig. 4C). After VIGS-treatments, the detached leaves of ‘White Heaven’ were inoculated with *B. cinerea* spores to observe disease symptoms. The *LlWRKY33*-silenced lily plants showed more severe necrotic disease symptoms than VIGS-controls (Fig. 4D and E). The further DAB staining analysis revealed that the *LlWRKY33-*silenced leaves had a higher H_2_O_2_ accumulation (Fig. 4F). The determination of H_2_O_2_ content also confirmed this result (Fig. 4G). These data implicated that silencing of *LlWRKY33* caused more *B. cinerea*-triggered ROS generation in lily. The results of trypan blue staining show that cell death caused by *B. cinerea* infection was more clearly visible at 24 hpi in *LlWRKY33*-silenced leaves than in VIGS-controls (Fig. 4H). Based on these results, it could be concluded that *LlWRKY33* confers *B. cinerea* resistance by alleviating cell death and the ROS accumulation.

**Figure 5:**
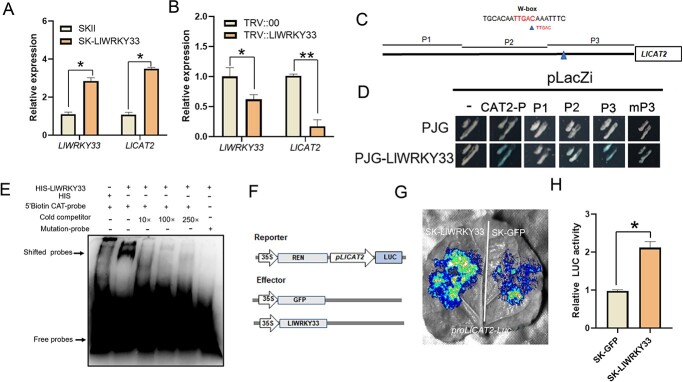
LlWRKY33 activates the expression of *LlCAT2*. **A** The expression of *LlWRKY33* and *LlCAT2* in *LlWRKY33*-overexpressing lily plants confirmed by RT-qPCR. Significant differences are presented as means ± SD of three replicates (Student’s *t* test, ^*^P < 0.05). **B** The expression of *LlWRKY33* and *LlCAT2* in *LlWRKY33*-silencing lily plants confirmed by RT-qPCR. Significant differences are presented as means ± SD of three replicates (Student’s *t* test, ^*^P < 0.05, ^**^P < 0.01). **C** The *LlCAT2* promoter and the W-box element marked with blue triangle. **D** Y1H assay for LlWRKY33 and the *LlCAT2* promoter. **E** EMSA of HIS-LlWRKY33 and the W-box element. **F** Schematic representation of the dual-luciferase reporter assay. **G** LUC signal in *N. benthamiana* leaves. Representative image was from one of three biological replicates. **H** Measurement of relative LUC activity. Significant differences are presented as means ± SD of three replicates (Student’s *t* test, ^*^P < 0.05).

### Overexpression of *LlWRKY33* enhances the resistance of transgenic Arabidopsis to *B. cinerea*

To further confirm the function of *LlWRKY33*, we transformed *LlWRKY33* into Arabidopsis to further determine its function after *B. cinerea* infection. Three *LlWRKY33*-transgenic lines (OE-2, OE-3, and OE-4) confirmed by RT-PCR (Fig. S6, see online supplementary material) were selected for further studies. To examine the defensive effect of *LlWRKY33* against *B. cinerea*, the transgenic Arabidopsis and wild-type were inoculated with *B. cinerea* using droplet-inoculation or spray-inoculation. The *LlWRKY33*-transgenic lines developed much milder symptoms than that of wild-type at 48 hpi (Fig. 4I and J; Fig. S7, see online supplementary material). Trypan blue staining assays also indicated that cell death was obviously inhibited in *LlWRKY33*-transgenic Arabidopsis compared with the wild-type (Fig. 4K), which suggested *LlWRKY33*-overexpression enhances the resistance of transgenic Arabidopsis to *B. cinerea*.

###  LlWRKY33 directly activates the expression of *LlCAT2* by binding its promoter

Because the accumulation of *LlWRKY33* in lily affects the levels of H_2_O_2_, we proposed that the expression of H_2_O_2_ scavenging enzyme genes may be activated by *LlWRKY33* after *B. cinerea* infection. To test this proposal, we tested the expression of several H_2_O_2_ scavenging enzyme genes such as *LlAPX2 (ASCORBATE PEROXIDASE 2)*, *LlCAT2*, *LlSOS1(SALT OVERLY SENSITIVE 1)*, *LlGPX8(GLUTATHIONE PEROXIDASE 8)*, and *LlSOD1(SUPEROXIDE DISMUTASE 1)* in lily plants. Transient overexpression of *LlWRKY33* activated the expression of *LlAPX2* and *LlCAT2* in leaves and transient silencing of *LlWRKY33* decreased their expression compared with that of control plants (Fig. 5A and B; Fig. S8, see online supplementary material). To further investigate the relationship between LlWRKY33 and *LlAPX2*, *LlCAT2*, we isolated the promoters of *LlAPX2* and *LlCAT2* and found that there were two W-box elements (−403 bp to −398 bp GTCAA; −286 bp to −281 GTCAA) on *LlAPX2* promoter and one W-box element (−223 bp to −218 bp TTGAC) on *LlCAT2* promoter (Fig. 5C; Tables S2 and S3, see online supplementary material). Further results of Y1H assay implicated that LlWRKY33 directly bound the *LlCAT2* promoter but not the *LlAPX2* promoter (Fig. 5D; Fig. S9, see online supplementary material). Next, we truncated the *LlCAT2* promoter into three fragments for Y1H assay and found that LlWRKY33 could only bind the P3 fragment, but not P1 or P2 (Fig. 5D). LlWRKY33 could not bind the mutant P3 fragment in which the TTGAC was mutated as TTTTT (Fig. 5D). Based on these results, the P3 region containing the W-box element was synthesized into a 5′ biotin-labeled probe for an EMSA. The result of EMSA was consistent with those Y1H assay (Fig. 5E). Additionally, the LUC activity of co-transformed with LlWRKY33 and *proLlCAT2*-LUC was stronger than that of the SK-GFP and *proLlCAT2*-LUC (Fig. 5G and H). All these results suggested that LlWRKY33 could directly activate the transcription of *LlCAT2*.

###  LlHSFA4 binds to the promoter of *LlCAT2* and activates its expression

**Figure 6:**
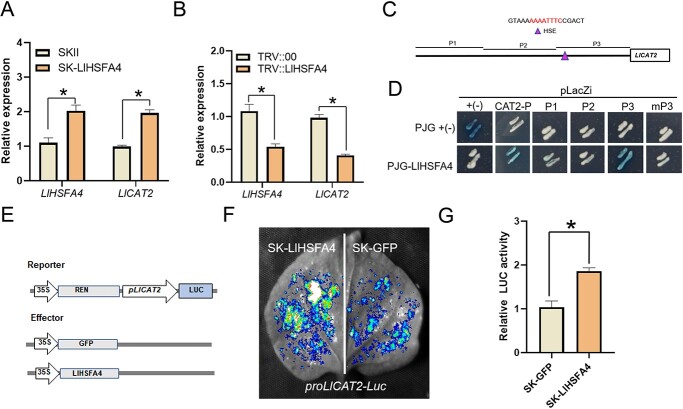
LlHSFA4 activates the expression of *LlCAT2*. **A** The expression of *LlHSFA4* and *LlCAT2* in *LlHSFA4*-overexpressing lily plants confirmed by RT-qPCR. Significant differences are presented as means ± SD of three replicates (Student’s *t* test, ^*^P < 0.05). **B** The expression of *LlHSFA4* and *LlCAT2* in *LlHSFA4*-silencing lily plants confirmed by RT-qPCR. Significant differences are presented as means ± SD of three replicates (Student’s *t* test, ^*^P < 0.05). **C***LlCAT2* promoter and the HSE marked with purple triangle. **D** Y1H assay for LlHSFA4 and the *LlCAT2* promoter. **E** Schematic representation of the dual-luciferase reporter assay. **F** The LUC signal in *N. benthamiana* leaves. Representative image was from one of three biological replicates. **G** Measurement of relative LUC activity. Significant differences are presented as means ± SD of three replicates (Student’s *t* test, ^*^P < 0.05). Three biological replicates were performed for each experiment.

Because silencing of *LlHSFA4* caused more H_2_O_2_ accumulation after *B. cinerea* infection, we then tested the expression of several H_2_O_2_ scavenging enzyme genes such as *LlAPX2*, *LlCAT2*, *LlSOS1*, *LlGPX8*, and *LlSOD1* in lily plants. The expression of *LlCAT2* was increased in transiently *LlHSFA4*-overexpression lily leaves, while silencing of *LlHSFA4* led to an opposite effect (Fig. 6A and B; Fig. S10, see online supplementary material). Then we proposed that LlHSFA4 might act as a regulator of *LlCAT2* because LlHSFA4 has the characteristic of a transcription factor which had transcriptional activation in yeast cells and localized in the nucleus (Fig. S11, see online supplementary material). We analysed the promoter of *LlCAT2* and found a potential HSE in the promoter of *LlCAT2* (Fig. 6C). The results of Y1H assay implicated LlHSFA4 bound the promoter of *LlCAT2* and only bind the P3, but not P1 or P2 (Fig. 6D). We also found that LlHSFA4 could not bind the mutant P3 (Fig. 6D). In addition, the luciferase reporter assay implicated that the LUC activity co-transformed with LlHSFA4 and *proLlCAT2*-LUC was stronger than that of the SK-GFP (Fig. 6F and G). Therefore, these data suggested that *LlCAT2* was directly regulated by LlHSFA4.

**Figure 7:**
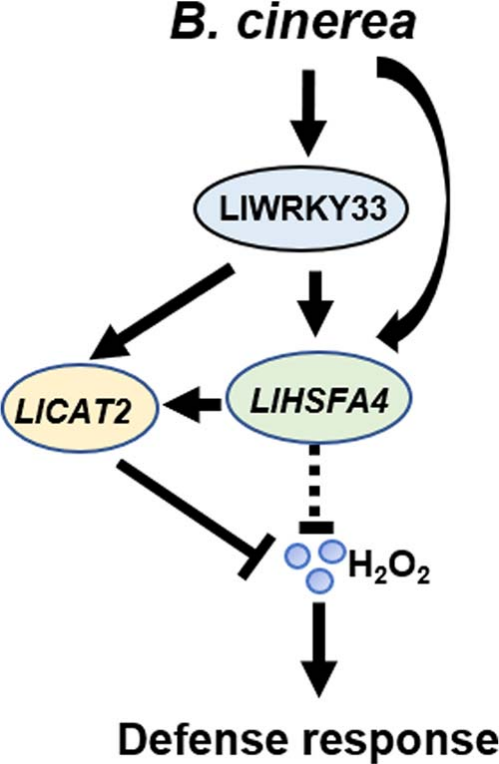
Working model of LlWRKY33-LlHSFA4-LlCAT2 module under *Botrytis cinerea* attack. When lily is infected with *B. cinerea*, *LlWRKY33* is rapidly induced and directly activate the expression of *LlHSFA4* and *LlCAT2* by binding to their promoters. Notably, *LlHSFA4* could also directly bind to the HSE on the promoter of *LlCAT2* and activate its expression. *LlCAT2*, a ROS scavenging enzyme, acts as a common target of LlWRKY33 and LlHSFA4 to prevent cell death caused by ROS accumulation to confer resistance to *B. cinerea* in lily.

## Discussion

HSFAs are mainly implicated in plant thermotolerance by directly regulating their target genes [26–28]. Previous studies have also shown that HSFAs (LlHSFA1, LlHSFA2, LlHSFA3, LlHSFA4) of lily are all involved in the heat stress response [29–33]. In this study, we characterized *LlHSFA4*, which was induced by *B. cinerea* (Fig. 1; Fig. S1, see online supplementary material). In addition, our results indicated that LlHSFA4 was a positive regulator in the response to *B. cinerea* (Fig. 2). Studies in different species have shown that HSFA4 may be involved in a variety of stresses by regulating ROS homeostasis. Arabidopsis *HSFA4A* was reported to response to oxidative stress by regulating the expression of ROS scavenging enzyme genes [34]. *OsHSFA4a* increased the Cd tolerance of rice by activating transcription of metallothionein gene [35]. overexpression of *Helianthus annuus HSFA9* enhanced the tolerance of transgenic tobacco to drought stress and oxidative stress [36]. Additionally, HSFA4 enhanced the tolerance of Arabidopsis and chrysanthemum (*Chrysanthemum morifolium*) to salt stress through increasing regulating the ROS homeostasis [14, 37]. LlHSFA4 increased thermotolerance of transgenic plants through reducing H_2_O_2_ accumulation and up-regulating the transcription of heat-related genes [33]. These studies implicated that the HSFA4 could response to many stresses such as Cd, oxidative or salt stress by mediating ROS homeostasis. Consistent with previous studies, our results also indicate that *LlHSFA4* acts as a positive regulator in the response to *B. cinerea* by directly binding the *LlCAT2* promoter (Fig. 6). These results suggest that HSFA4 may play a role in the plant’s response to biotic and abiotic stresses by regulating the ROS homeostasis.

Additionally, the several W-box elements were found in the promoter of *LlHSFA4* (Table S1, see online supplementary material). It was found that, among the six typical W-boxes, only the W-box 6 appeared to be bound by LlWRKY33 (Fig. 3), which implicated that the binding of LlWRKY33 to the W-box on the promoter of *LlHSFA4* was selective. This result can be explained by a previous study on WRKY TF binding site preferences, which depended in part on the sequences flanking W-box elements [38]. Several studies have also found that the WRKY33 is an important regulator in the response to heat stress in addition to its involvement in the response to *B. cinerea*. In Arabidopsis, AtWRKY33 positively regulate the heat stress response by coordinately working with AtWRKY25, AtWRKY26 [39]. Overexpression of *SlWRKY33* can restore the tolerance of Arabidopsis *wrky33* mutant plants to heat stress [23, 40]. Overexpression of *Triticum aestivum WRKY33* enhances the tolerance of Arabidopsis to heat stress [41]. Although WRKY33 and HSFA4 have been reported in the response to the heat stress and *B. cinerea* in plants, their regulatory mechanism between them is unclear. In this study, we demonstrated that *LlHSFA4* is a direct target of LlWRKY33 (Fig. 3), which indicated that LlWRKY33 and LlHSFA4 were closely related in the response to these two stresses. However, whether the relationship between WRKY33 and HSFA4 is conserved in different plants requires further research.

In natural conditions, plants will encounter a variety of environmental stresses. Sometimes, plant may subject to one or combinations of two or more stress simultaneously because of the variability of the living environment [42, 43]. Pathogen attacks and temperature stress are one of the most frequently combinations of stress [44–46]. Previous studies have also revealed that temperature affects plant-pathogen interactions by affecting disease resistance (*R*) genes effectiveness and durability or interfering with conidial germination and mycelial growth [47, 48]. *B. cinerea* occurs at low temperature and high humidity, which is most active at 23°C and relative humidity above 80% in tomato [49]. When the temperature rises above 30°C, the virulence of *B. cinerea* will be weakened [50, 51]. This could be explained that high temperature increases the expression of defense genes, which induces the plant’s resistance to *B. cinerea* [52]. More reports have also shown that high temperature pretreatment has a direct positive effect on the host immunity, thereby improving their disease resistance [53–55]. A recent study has shown that LlHSFA4 plays a positive role in the establishment of basal thermotolerance [33]. In our study, LlHSFA4 confers resistance to *B. cinerea* (Fig. 2). These results suggest that LlHSFA4 may be potential linker in the response to heat stress and *B. cinerea* infection. However, the specific mechanism needs to be confirmed by further studies.

In this study, we identified the roles of *LlHSFA4* and *LlWRKY33* following inoculation with *B. cinerea*. Additionally, *LlHSFA4*, as a direct target of LlWRKY33, together with *LlWRKY33* activates *LlCAT2* to regulate ROS homeostasis (Fig. 7), thereby improving the resistance of lily to *B. cinerea*.

## Materials and methods

### Plant materials and growth conditions

The plant materials, lily hybrid ‘White heaven’ (*L. longiflorum*), tobacco (*N. benthamiana*) and *Arabidopsis thaliana* (Col-0) were grown at 22°C in a culture room (16-h light/8-h dark).

### Protein localization and transcriptional activity analysis

The specific primers for genes cloning were shown in a list (Table S4, see online supplementary material). The pCAMBIA1300-GFP vector was used to construct LlWRKY33-GFP. The culture containing the pCAMBIA1300-LlWRKY33 plasmid was collected and resuspended in the buffer as previously described [56]. The resuspended buffer was injected into *N*. *benthamiana* leaves. The GFP signal was observed by a laser scanning confocal microscope (LSM800, Zeiss, Germany). The pGBKT7 vector was used to construct pGBKT7-LlWRKY33 and pGBKT7-LlHSFA4. The vectors of pGBKT7 and constructed vector were introduced into AH109 yeast cells and screened on SD media (lacking Trp and His).

### Pathogen inoculation

*B. cinerea* strain B05.10 [57] was cultured on potato dextrose agar (PDA) medium for two weeks for sporulation. The *B. cinerea* spores were resuspended in sterile distilled water and collected by centrifugation and then adjusted to a concentration of 10^6^ spores/ml with half-strength potato dextrose broth (PDB). For inoculation, the leaves of lily were chosen to be inoculated with 20 μL of the *B. cinerea* spores by droplet-inoculation. After inoculation of 60 h, phenotypes were photographed by a camera and lesion diameter of at least 20 infected leaves each time were determined using Image J software. The rosette leaves of one-month-old transgenic Arabidopsis and wild-type were chosen to be inoculated with 5 μL of the *B. cinerea* spores. After inoculation of 48 h, lesion diameter of at least 10 infected rosette leaves each time were determined using Image J software. All experiments were repeated three times.

### Promoter isolation and GUS activity assay

All promoter sequences used in this study were obtained by the hiTAIL-PCR method [58]. All promoter were analysed by the databases New PLACE (https://www.dna.affrc.go.jp/PLACE/?action=newplace). GUS staining was performed as previously described [59].

### Gene expression assay

12-mm diameter samples detached from ‘White Heaven’ leaves with a puncher were chosen to be inoculated with 20 μL of the *B. cinerea* spores by droplet-inoculation. Then, the treated leaves were put on sterile filter paper and kept moist in a plastic dish. The treated leaves were collected at 0, 3, 6, 12, 24, 36, 48, and 60 hpi. RT-qPCR was performed with SYBR Premix Ex Taq II system (Vazyme, Nanjing, China) on the Quant Studio 6 Flex (ABI, Shanghai, China). Data was analyzed with the 2^-∆∆Ct^ method [60]. The lily 18S *rRNA* was used as the endogenous gene. Three independent technical replicates were performed for each of three biological replicates.

### Virus-induced gene silencing in lily

*Agrobacterium*-mediated infection of TRV-based VIGS assay [61] was used to silence gene expression in lily as described by our previous study [62]. The 293-bp fragment of *LlWRKY33* and 269-bp fragment of *LlHSFA4* were introduced into the pTRV2 vector. The pTRV1, pTRV2, and the reconstructed vectors were transformed into *Agrobacterium tumefaciens* strain GV3101, individually. *Agrobacterium* culture containing TRV1 and TRV2 at a ratio of 1:1 was co-infiltrated into the leaves of lily. Then the infiltrated lily plants were put in dark conditions for 1 day and a culture room for 4 days. Then we collected 5-day leaves with a hole punch (12 mm in diameter) for inoculation to observe the disease symptoms or RT-qPCR analysis to detect the gene silencing efficiency.

### Transient overexpression assay in lily

*LlWRKY33* and *LlHSFA4* were respectively introduced into the SK-II vector. *Agrobacterium* cultures containing SK-II, SK-LlWRKY33, or SK-LlHSFA4 were respectively injected into the leaves of lily. The 3-day infiltrated leaves were selected for RT-qPCR.

### Histochemical staining

Inoculated leaves were stained for histological assay by DAB or trypan blue as previously described [63, 64]. The accumulation of H_2_O_2_ was observed with DAB staining (1 mg/ml) and destained with 95% ethanol. The H_2_O_2_ content was determined with a H_2_O_2_ Content Assay Kit (Sangon Biotech, Shanghai, China). Trypan blue staining (0.04%) was used to detect cell death and destained with saturated chloral hydrate solution (2.5 g·mL^−1^).

### Generation of transgenic Arabidopsis and phenotypic analysis

The bacterial solution of pCAMBIA1300-LlWRKY33 and pCAMBIA1300-LlHSFA4 were collected by centrifugation and resuspended in sucrose solution (5%). The resuspended bacterial solutions were infiltrated *A. thaliana* (Col-0) using the flower-dipping method [65] to generate transgenic Arabidopsis lines. The T3 generations of transformants were selected to perform the phenotypic analysis. To compare the resistance to pathogen of transgenic lines and wild type, the detached rosette leaves of transgenic lines and wild type were treated with *B. cinerea* spores. After 48 h, phenotypes were recorded.

### Yeast one-hybrid assay(Y1H)

For Y1H assays, the ORF of *LlWRKY33* and *LlHSFA4* were respectively inserted into pB42AD plasmid (Clontech, CA, USA). The promoters of *pLlHSFA4*, *pLlCAT2*, and *pLlAPX2* were respectively inserted into pLacZ vector (Clontech, CA, USA). Recombinant and empty vectors were introduced into EGY48a as described by a previous study [66], and cultured on the SD medium lacking Trp, Ura medium at 30°C.

### Luciferase reporter assay

The isolated promoter fragments of the *LlHSFA4* and *LlCAT2* were fused into pGreenII0800-LUC and the ORF of *LlWRKY33* and *LlHSFA4* were inserted into a pGreenII62-SK-GFP as described by our study [67].The resuspended bacterial solutions containing the effector plasmid and the reporter plasmid were injected into *N. benthamiana* leaves. The relative LUC activity was quantified using Image J v1.8.0 software.

### Electrophoretic mobility shift assay (EMSA)

The pET32a vector was used to generate HIS-LlWRKY33 fusion protein. Target promoter region and mutant target region of *LlHSFA4* and *LlCAT2* were labeled with biotin by TSINGKE Biological Technology (Nanjing, China). Unlabeled DNA of the same sequence was used as a competitor. The EMSA assay was carried out with an EMSA kit (Thermo Fisher, New York, NY, USA). The signal was detected by a CCD camera (Tanon 5200, Shanghai, China).

## Acknowledgements

This research was supported by the National Key R&D Program of China (2023YFD2300900), the Project for Crop Germplasm Resources Conservation of Jiangsu (2021-SJ-011), and the Modern Agricultural Industry Technology System in Jiangsu [JATS (2023) 007].

## Author contributions

N.T. and Z.W. designed the research; L.D. and Z.W. conducted the experiments and data processing under the supervision of N.T.; L.D. wrote the manuscript; J.X. S.X., Y.Z., and D.Z. provided technological assistance; X.C. isolated and provided strains of *Botrytis cinerea*. All authors read and revised the article.

## Data availability

The data and figures in this study can be found within the article and its supporting materials.

## Conflict of interest statement

All authors state that they have no conflict of interest in relation to this research.

## Supplementary data

Supplementary data is available at *Horticulture Research* online.

## Supplementary Material

Web_Material_uhad254
