# Supplementary material for: A LlWRKY33-LlHSFA4-LlCAT2 module confers resistance to Botrytis cinerea in lily
Source: Hortic Res. 2023 Nov 27;11(1):uhad254. doi: 10.1093/hr/uhad254 (PMC10809907; doi:10.1093/hr/uhad254)
Supplement: Web_Material_uhad254 [file web_material_uhad254.zip › Supplementary Figures.docx]

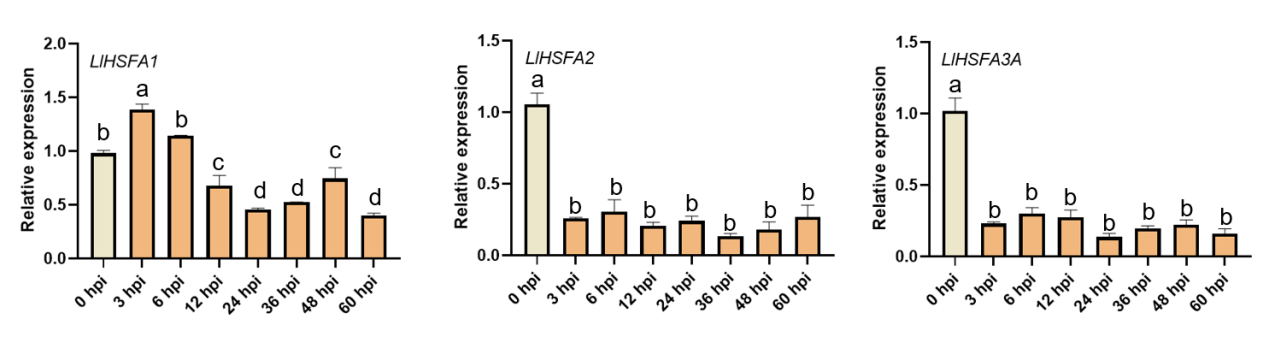


Supplementary Fig. 1 Relative expression levels of *LlHSFA1*, *LlHSFA2*, *LlHSFA3A* in lily plants infected with *B*. *cinerea* at various hours post-inoculation confirmed by RT-qPCR. Lily *18S rRNA* was used as an endogenous gene. Different letters indicate significant differences determined by Tukey’s test with the standard deviation. (P < 0.05).


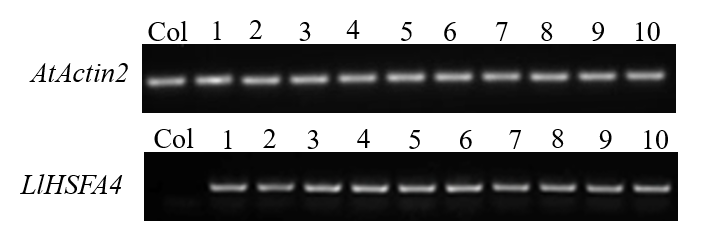


Supplementary Fig. 2 RT-PCR analysis of *LlHSFA4* transgenic Arabidopsis lines. One-week-old seedlings were used to detect the expression of *LlHSFA4* in T3 transgenic Arabidopsis lines by RT-PCR. *AtActin2* was used as an endogenous gene. The number of PCR cycles is 28 and the template was 1 µL.


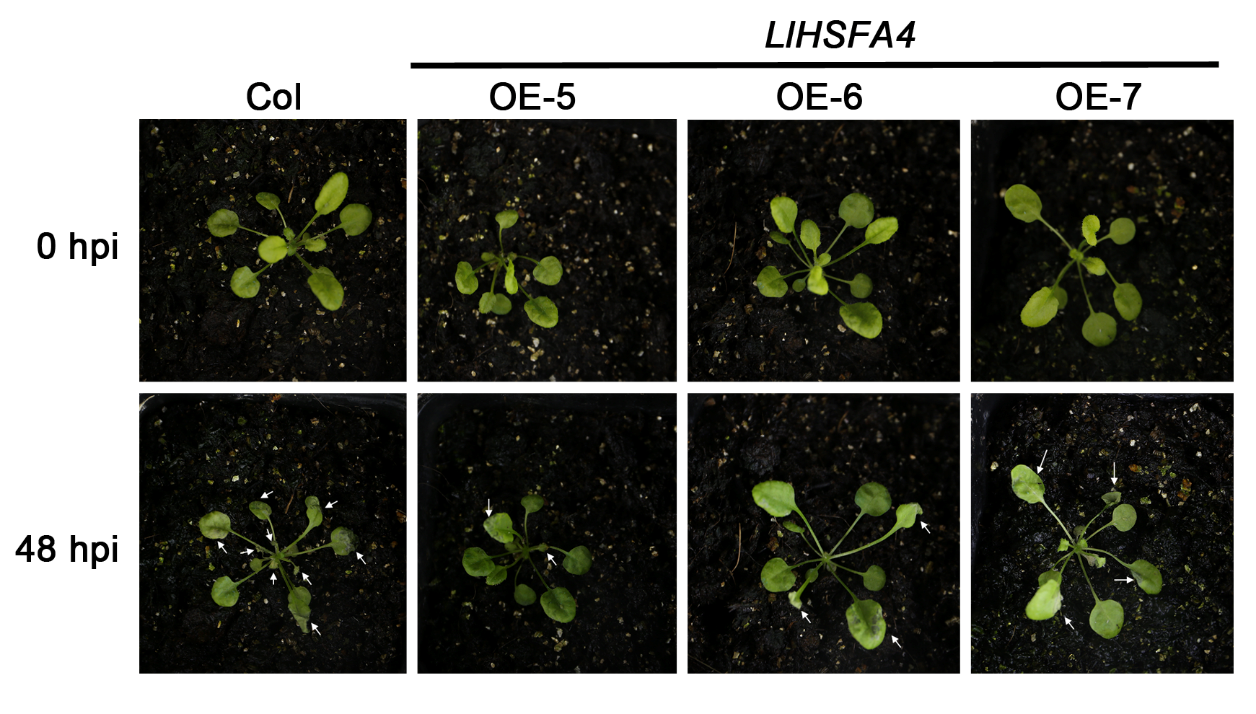


Supplementary Fig. 3 Representative image of phenotype on the wild-type and three *LlHSFA4*-transgenic lines spray-inoculated with *B. cinerea* spores at 0 hpi and 48 hpi. The arrows indicate disease symptoms.


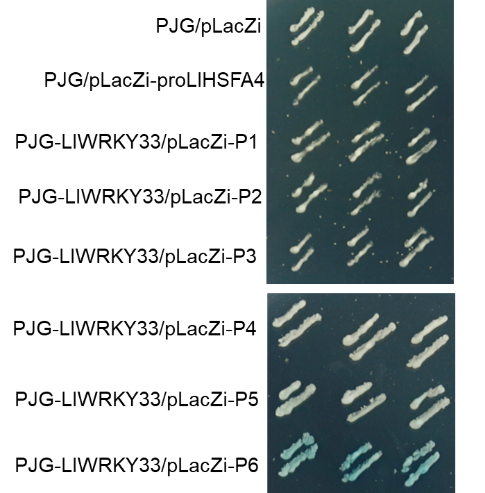


Supplementary Fig. 4 A Y1H assay for LlWRKY33 and the fragments of *LlHSFA4*.


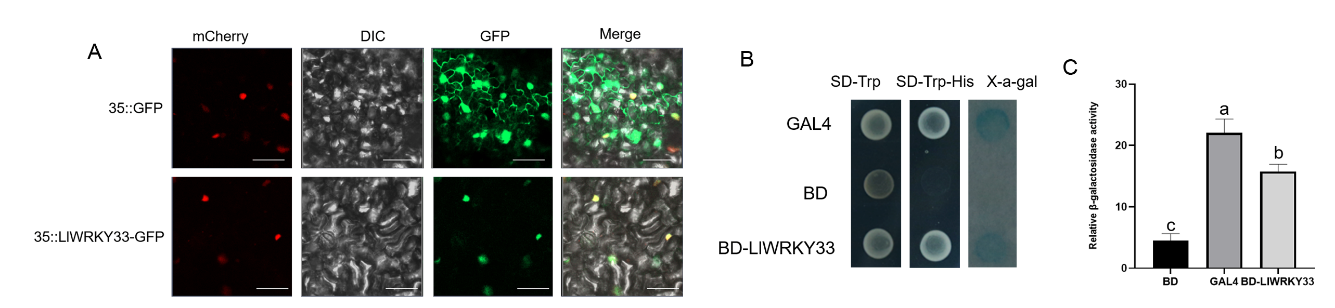


Supplementary Fig.5 Subcellular localization and transactivation assay of LlWRKY33. A Detection of fluorescence signals in *N. benthamiana* co-transfected with LlWRKY33-GFP and the nuclear marker RFP-NLS. Scale bar=50 μm. B Transactivation activity assay in the yeast AH109 strain. Representative image based on three replicates. C Determination of β-galactosidase activity. Data are presented as means ± SD of three clones.


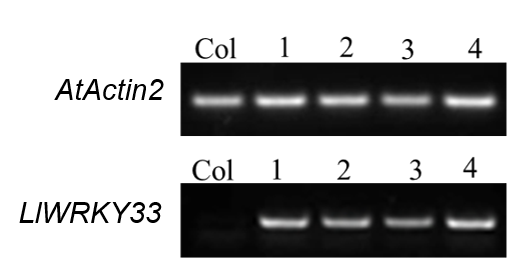


Supplementary Fig. 6 RT-PCR analysis of *LlWRKY33* transgenic Arabidopsis lines. One-week-old seedlings were used to detect the expression of *LlWRKY33* in T3 transgenic Arabidopsis lines by RT-PCR. *AtActin2* was used as an endogenous gene. The number of PCR cycles is 28 and the template was 1 µL.


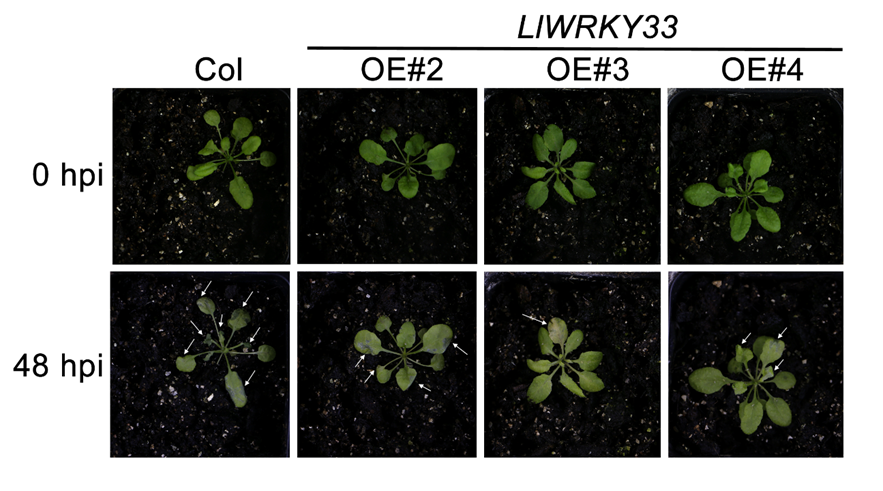


Supplementary Fig. 7 Representative image of phenotype on the wild-type and three *LlWRKY33*-transgenic lines spray-inoculated with *B*. *cinerea* spores at 0 hpi and 48 hpi. The arrows indicate disease symptoms.


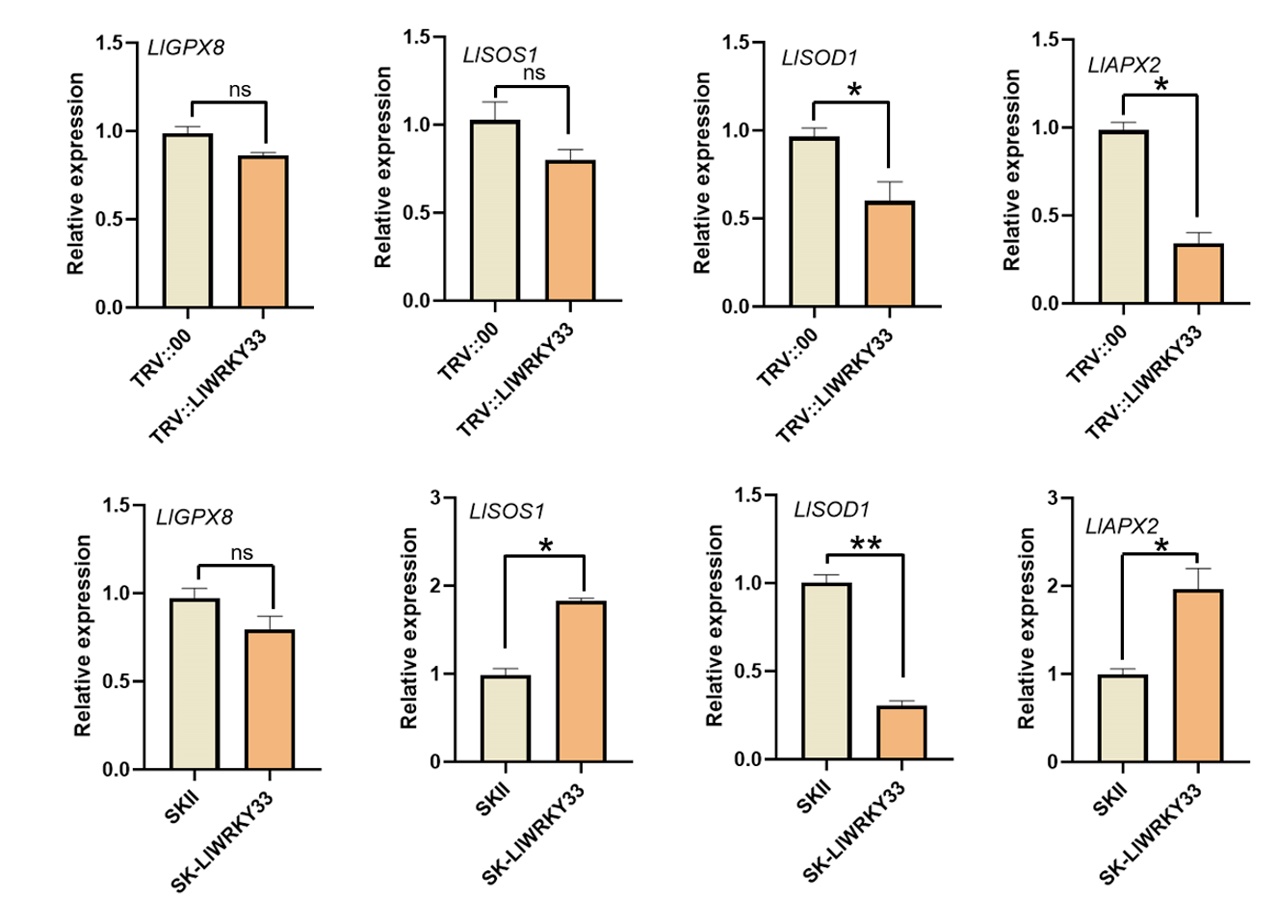


Supplementary Fig. 8 Relative expression levels of H_2_O_2_ scavenging enzyme genes in *LlWRKY33*-silencing and *LlWRKY33*-overexpressing lily plants. Lily *18S rRNA* was used as an endogenous gene. Significant differences are presented as means ± SD of three replicates (Student’s *t*-test, *P < 0.05, **P < 0.01) ns: not significant.


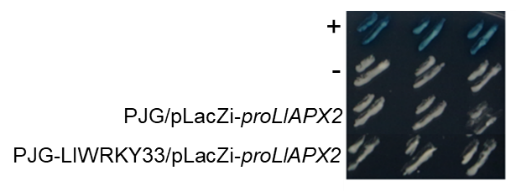


Supplementary Fig. 9 A Y1H assay for LlWRKY33 and the promoter of the *LlAPX2*. Representative image based on three replicates.


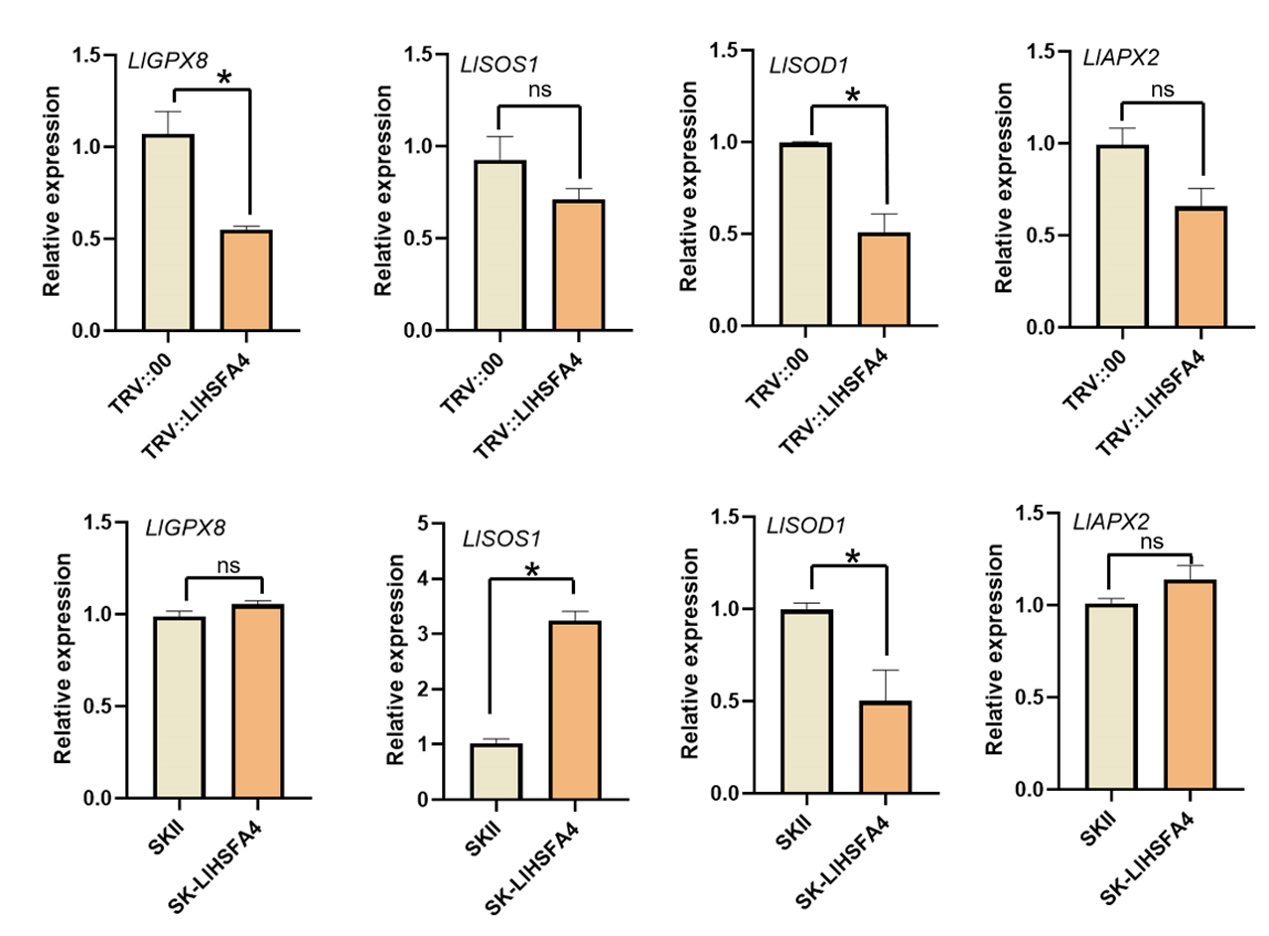


Supplementary Fig.10 The expression level of other ROS-scavenging enzyme genes in *LlHSFA4*-silencing and *LlHSFA4*-overexpressing lily plants confirmed by RT-qPCR. Significant differences are presented as means ± SD of three replicates (Student’s *t*-test, *P < 0.05) ns: not significant.


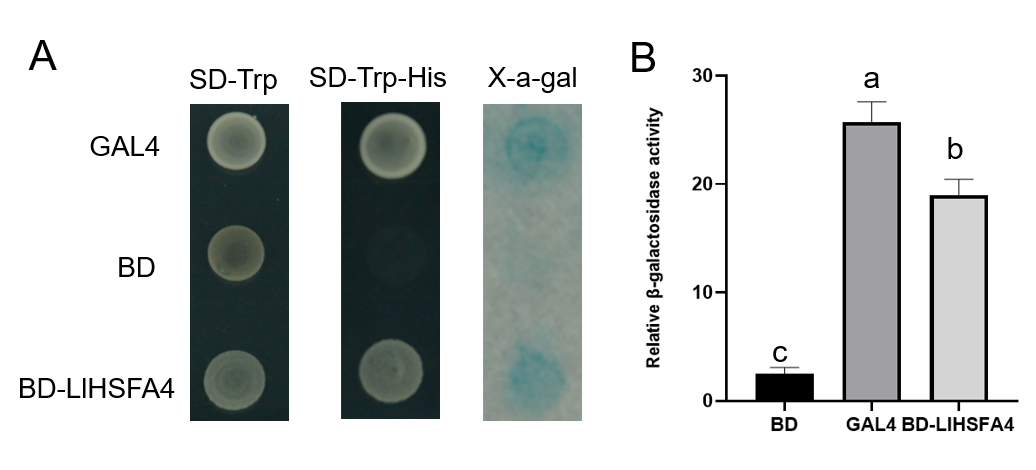

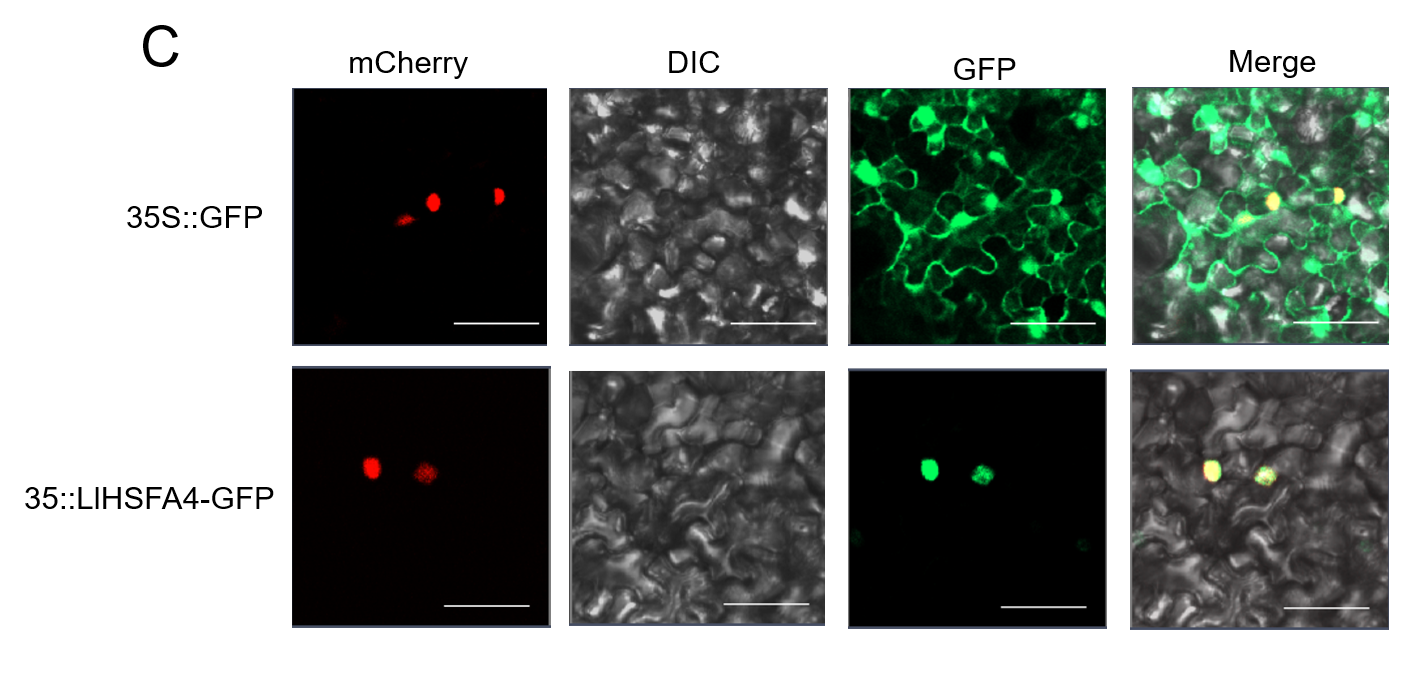


Supplementary Fig. 11 Transactivation assay and subcellular localization of LlHSFA4. A Transactivation activity assay in the yeast AH109 strain. Representative image based on three replicates. B Determination of β-galactosidase activity. Data are presented as means ± SD of three clones. C Detection of fluorescence signals in *N. benthamiana* co-transfected with LlHSFA4-GFP and the nuclear marker RFP-NLS. Scale bar=50 μm.
