# Supplementary figures and images for: A LlWRKY33-LlHSFA4-LlCAT2 module confers resistance to Botrytis cinerea in lily
Source: Hortic Res. 2023 Nov 27;11(1):uhad254. doi: 10.1093/hr/uhad254 (PMC10809907; doi:10.1093/hr/uhad254)

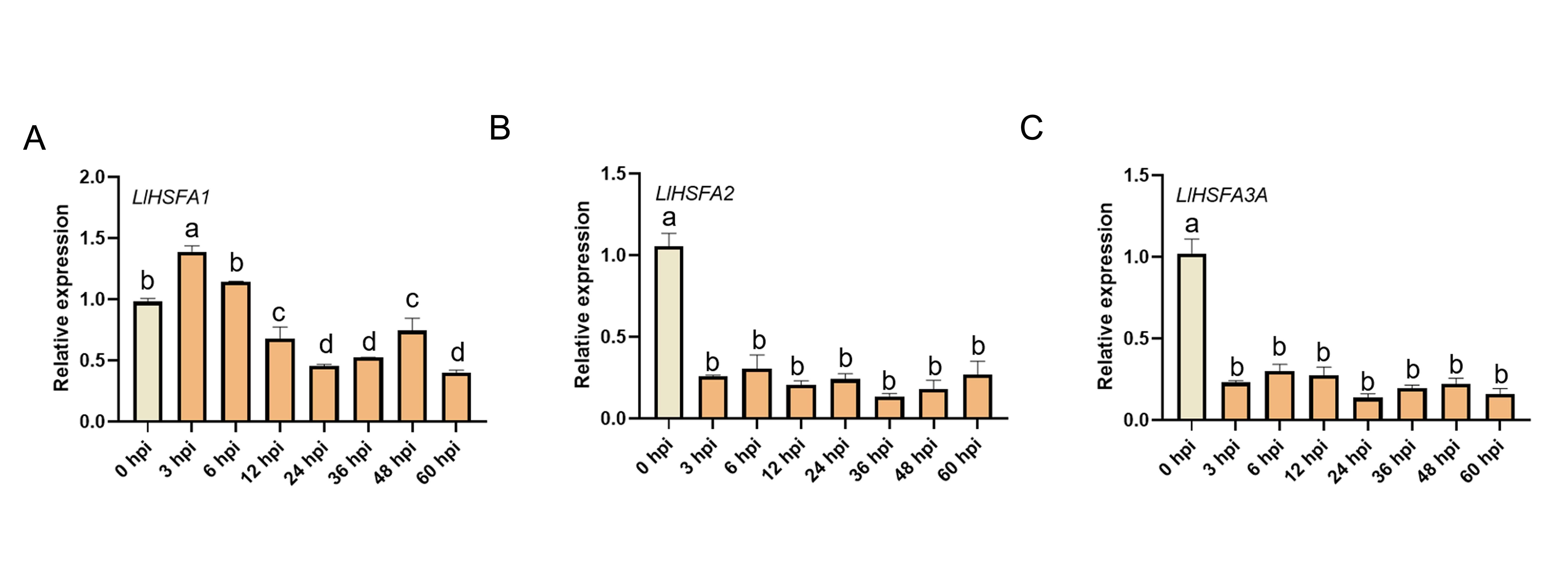

Supplement: Web_Material_uhad254 [file web_material_uhad254.zip › FigS1.jpg]

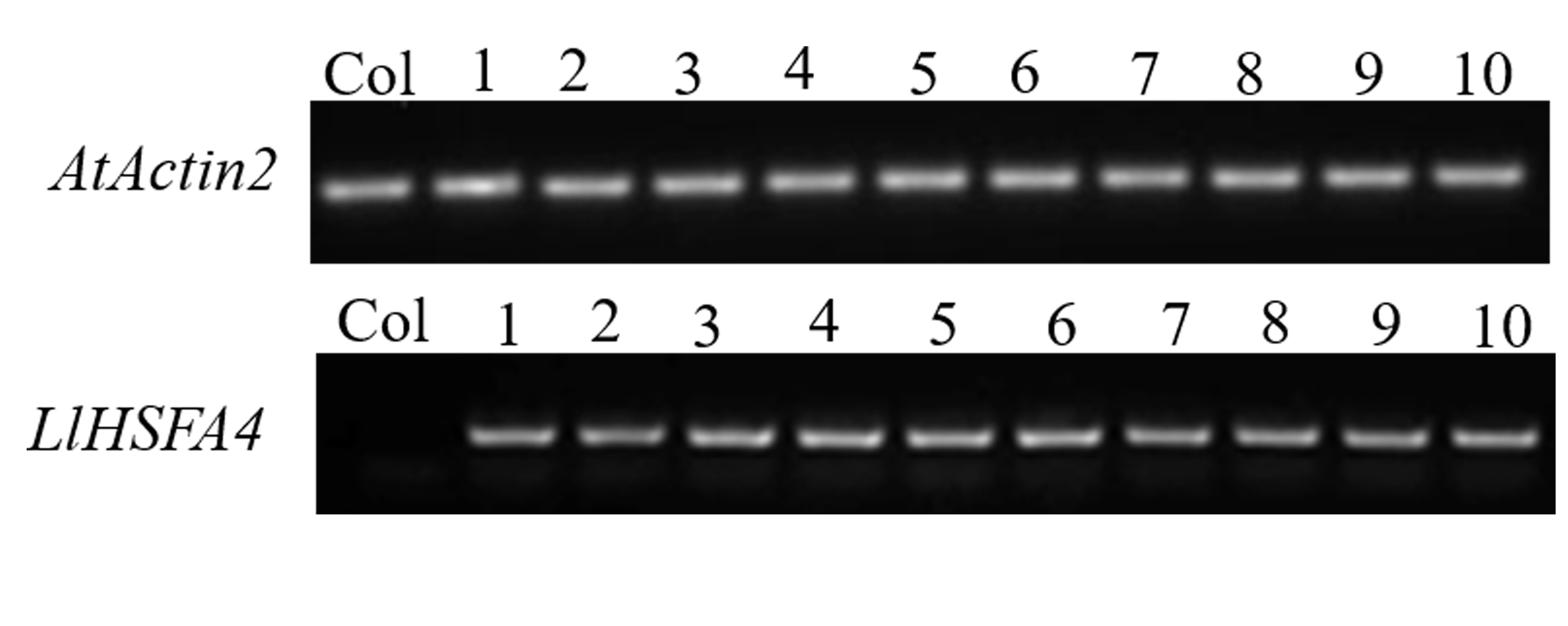

Supplement: Web_Material_uhad254 [file web_material_uhad254.zip › FigS2.jpg]

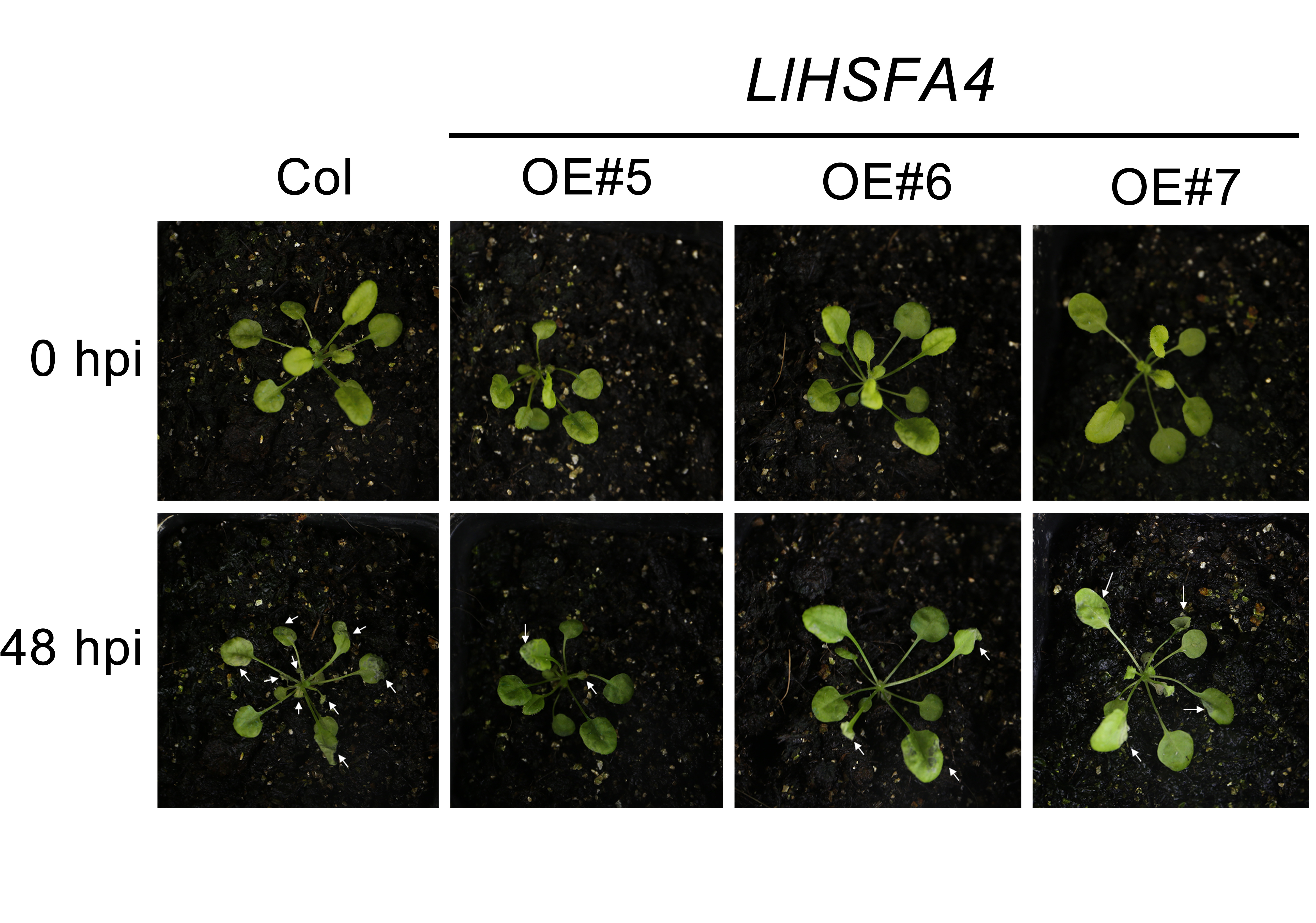

Supplement: Web_Material_uhad254 [file web_material_uhad254.zip › FigS3.jpg]

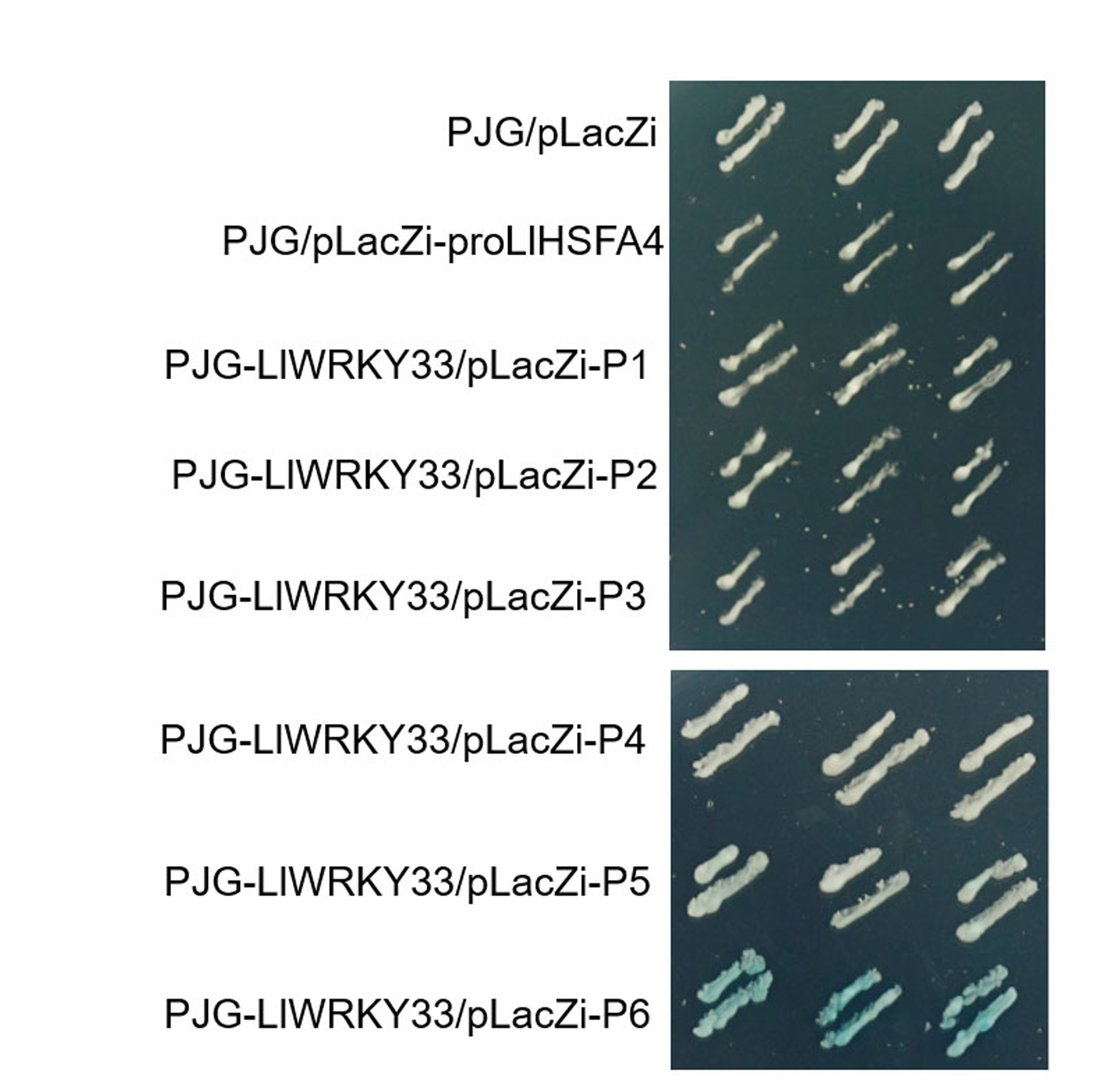

Supplement: Web_Material_uhad254 [file web_material_uhad254.zip › FigS4.jpg]

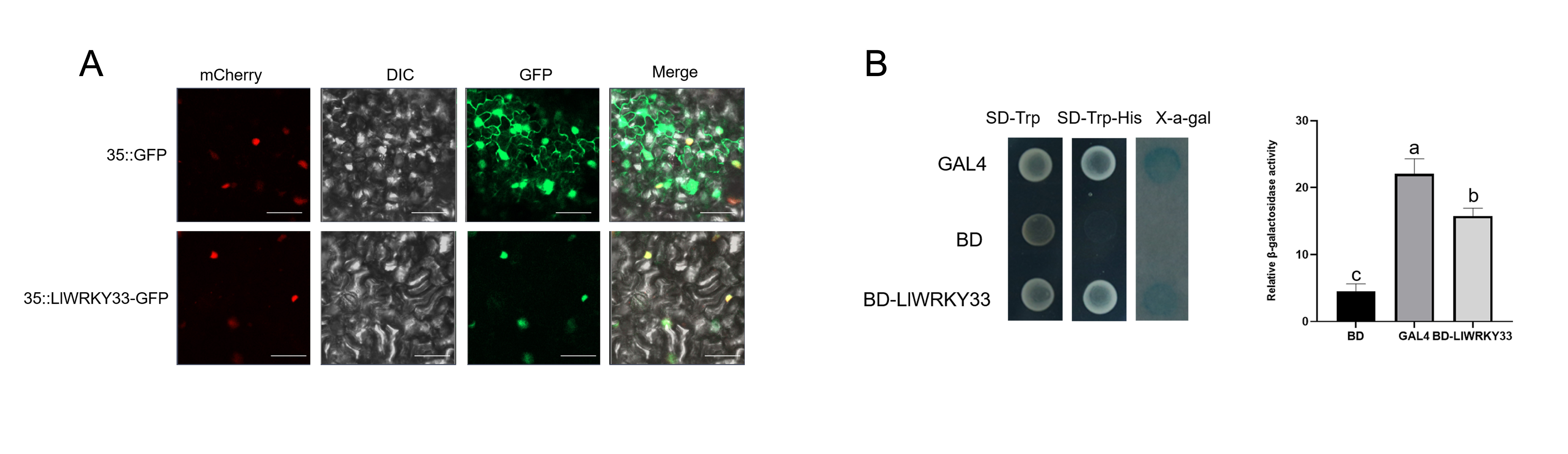

Supplement: Web_Material_uhad254 [file web_material_uhad254.zip › FigS5.jpg]

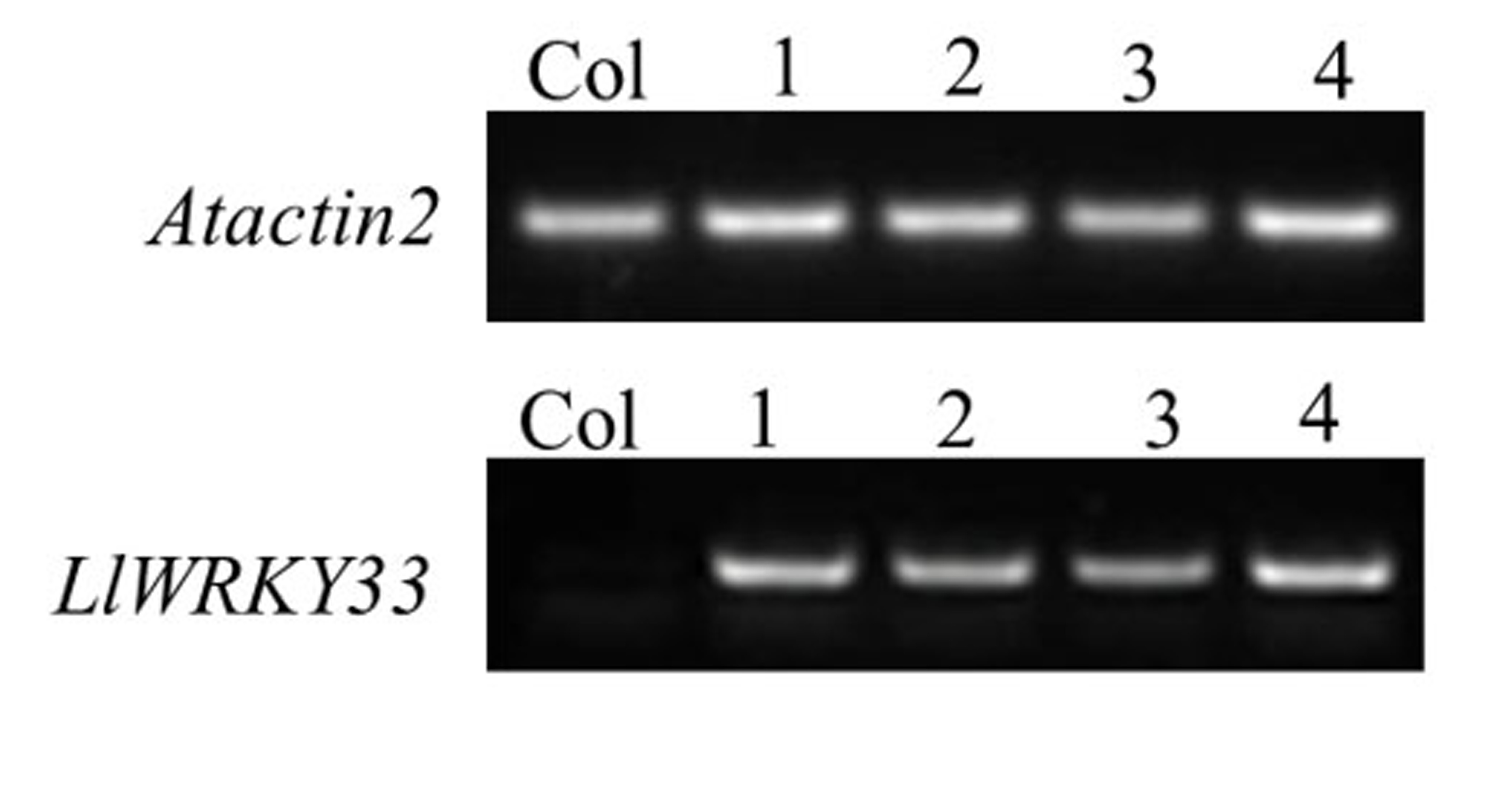

Supplement: Web_Material_uhad254 [file web_material_uhad254.zip › FigS6.jpg]

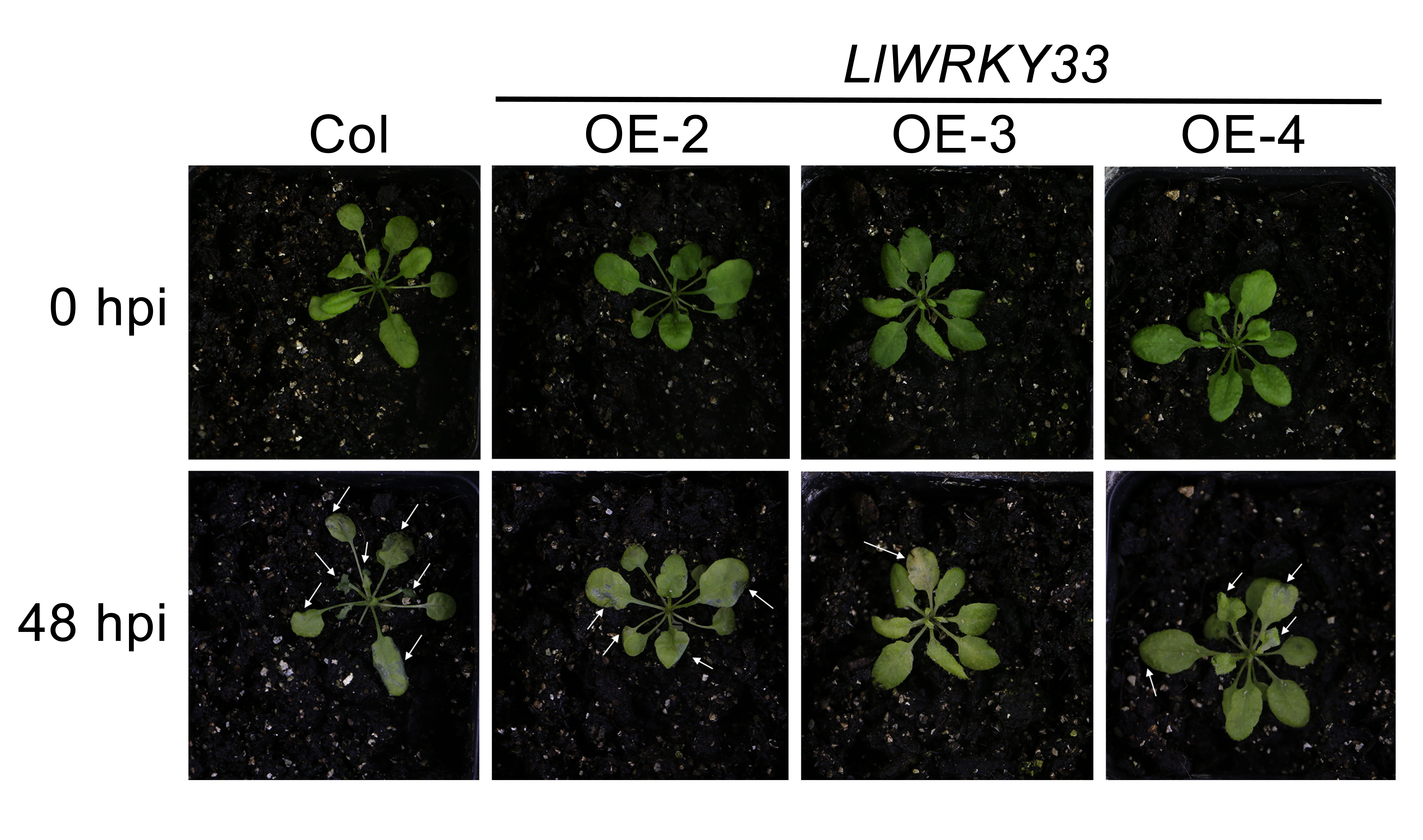

Supplement: Web_Material_uhad254 [file web_material_uhad254.zip › FigS7.jpg]

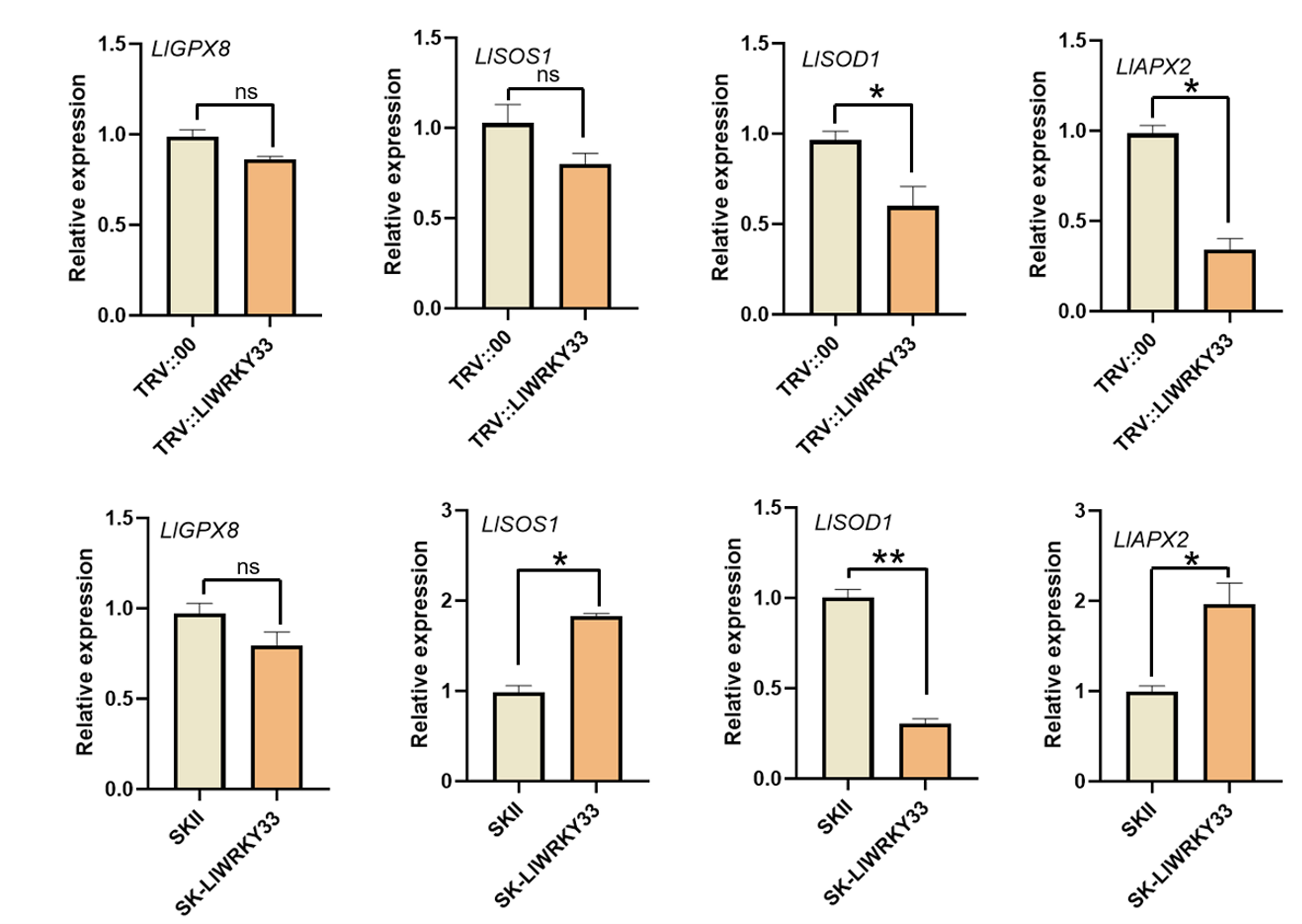

Supplement: Web_Material_uhad254 [file web_material_uhad254.zip › FigS8.jpg]

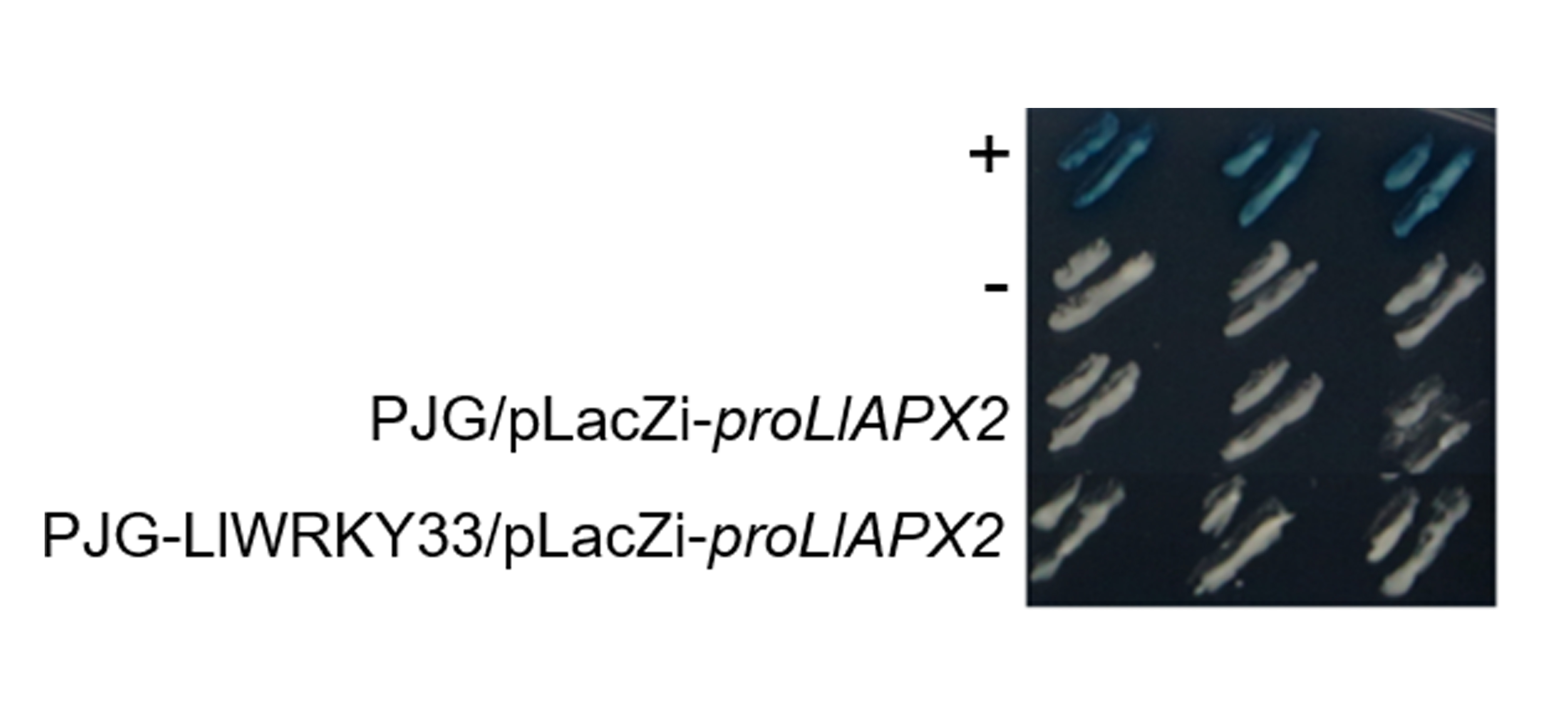

Supplement: Web_Material_uhad254 [file web_material_uhad254.zip › FigS9.jpg]

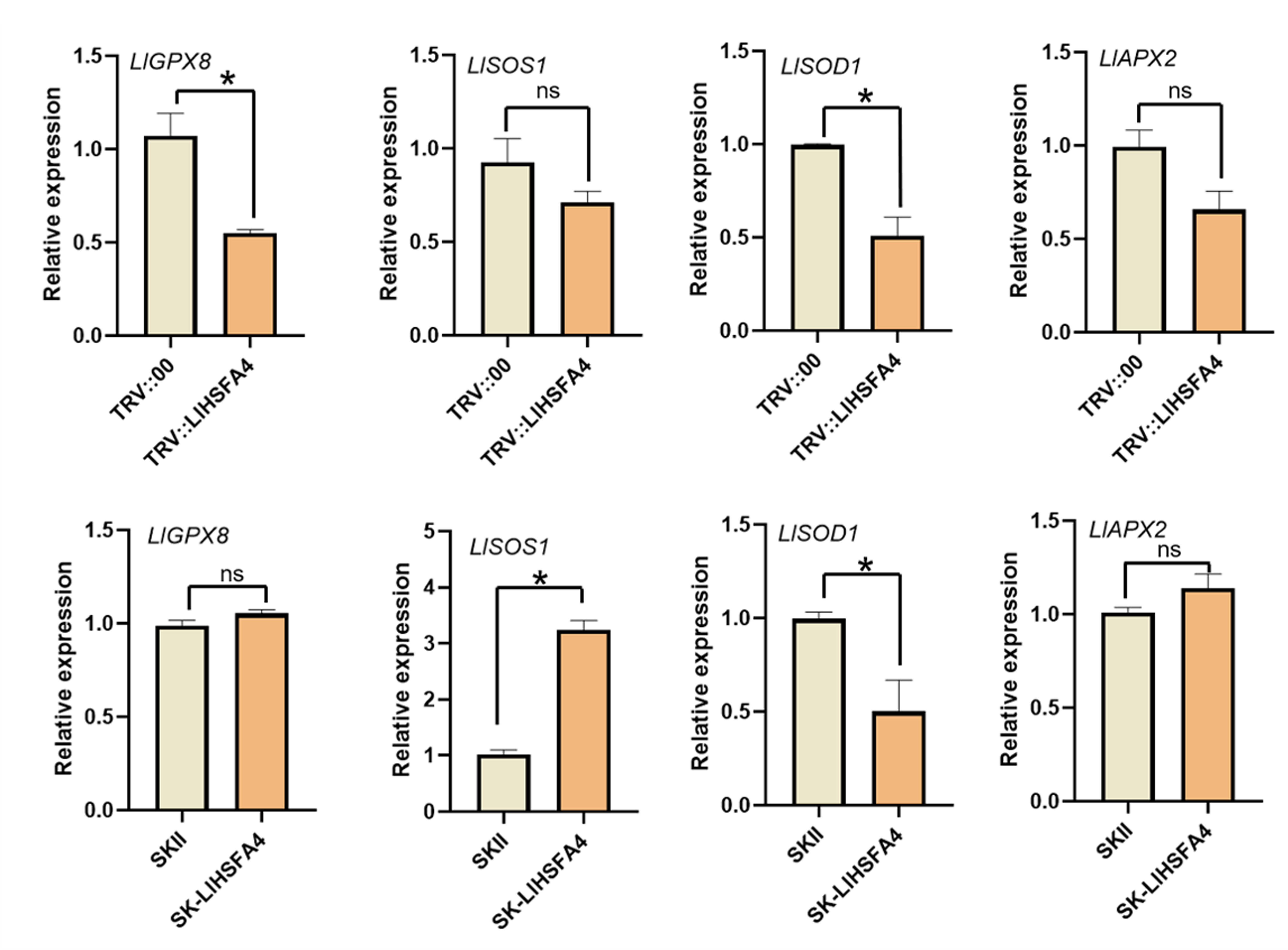

Supplement: Web_Material_uhad254 [file web_material_uhad254.zip › FigS10.jpg]

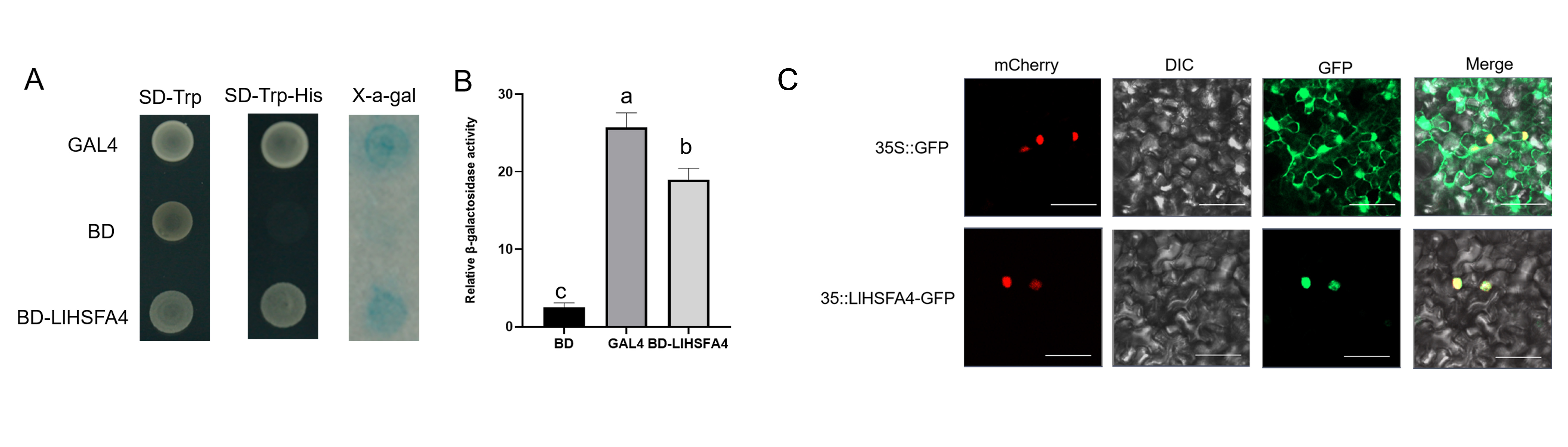

Supplement: Web_Material_uhad254 [file web_material_uhad254.zip › FigS11.jpg]
